# Analysis of co-occurrence of type II toxin–antitoxin systems and antibiotic resistance determinants in *Staphylococcus aureus*

**DOI:** 10.1128/msystems.00957-24

**Published:** 2025-02-27

**Authors:** Michał Bukowski, Michał Banasik, Kinga Chlebicka, Katarzyna Bednarczyk, Emilia Bonar, Dominika Sokołowska, Tomasz Żądło, Grzegorz Dubin, Benedykt Władyka

**Affiliations:** 1 Department of Analytical Biochemistry, Faculty of Biochemistry, Biophysics and Biotechnology, Jagiellonian University in Krakow, Krakow, Poland; 2 Doctoral School of Exact and Natural Sciences, Jagiellonian University in Krakow, Krakow, Poland; 3 Malopolska Centre of Biotechnology, Jagiellonian University in Krakow, Krakow, Poland; London School of Hygiene & Tropical Medicine, London, United Kingdom

**Keywords:** toxin–antitoxin system, *Staphylococcus*, *Staphylococcus aureus*, antibiotic resistance, mobile genetic elements, genome analysis

## Abstract

Toxin–antitoxin (TA) systems consist of toxic proteins and their inhibitors, and were originally shown to ensure plasmid maintenance in bacterial populations. Over time, however, TA systems have also been identified on bacterial chromosomes, raising questions about their roles unrelated to plasmid stability. Among the eight currently recognized types of TA systems, type II has been the most extensively investigated. Type II systems are often found in pathogenic bacterial species, including staphylococci. *Staphylococcus aureus*, a notorious human pathogen, harbors multiple type II TA systems, both plasmid- and chromosome-encoded, while their potential relation to virulence remains to be addressed. Here, we investigate the co-occurrence of TA systems and antibiotic resistance (AR) determinants in *S. aureus*, focusing on the potential negative impact of type II toxin RNases on antibiotic resistance. We considered both well-characterized and newly characterized TA loci of *S. aureus*. Our findings demonstrate a relationship between TA systems and AR determinants, wherein TA systems negatively affect antibiotic resistance. Due to substantial selective pressure, the migration of TA systems from plasmids to chromosomes results in their inactivation. This observation may be an important factor shaping the spread and evolution of both TA systems and AR determinants in bacteria. We exemplify this phenomenon in detail using the well-known PemIK-Sa1 system and a newly identified SCCmec-related PemIK-Sa6 system characterized in this study.

**IMPORTANCE:**

Toxin–antitoxin (TA) systems are entities unique to bacteria. They are involved in the maintenance of mobile genetic elements (MGEs), regulation of gene expression and bacterial virulence. *Staphylococcus aureus* is a dangerous human pathogen with increasing antibiotic resistance (AR). The maintenance and dissemination of AR determinants is often driven by MGEs, which link AR and TA systems. Our study identified a negative correlation between TA systems and AR determinants in *S. aureus*. Furthermore, we have shown that the expression of a toxic component of an exemplary TA system negatively affects antibiotic resistance. We argue that in particular strains, a selective pressure maintains either the TA system or AR determinant. Alternatively, TA systems are inactivated by mutations when present together with AR determinants to maintain the functionality of the latter. Our observations uncover an important factor shaping the spread and evolution of both TA systems and AR determinants in bacteria, which is especially relevant to pathogenic species.

## INTRODUCTION

Toxin–antitoxin (TA) systems are genetic elements coding for a toxic protein (toxin) and an inhibitor (antitoxin) of the toxin’s expression or activity (1). Initially discovered as components of plasmids, these systems were thought to function as plasmid-addiction modules (2). The mechanism, historically termed “post-segregational killing” (PSK), relies on the elimination of daughter cells that fail to inherit the plasmid. In the presence of the plasmid, both components of the TA system are expressed, preventing the toxin’s activity. However, upon plasmid loss, the labile antitoxin is rapidly degraded, allowing the more stable toxin to exert its deleterious effects. This cell poisoning or growth inhibition creates a significant disadvantage, thereby restricting the proliferation of plasmid-free daughter cells (3–5).

The association with mobile genetic elements (MGEs) plays a key role in the diversity and widespread distribution of TA systems among bacteria. MGEs serve as reservoirs of accessory genes that augment the core genome, imparting numerous advantages for bacterial fitness. This relationship is particularly pertinent in pathogens, where MGEs are known to harbor genes encoding virulence factors and antibiotic resistance (AR) determinants. Therefore, exploring the correlations between TAs and AR is crucial for understanding these dynamics.

For years, TA systems have been identified not only on plasmids and other MGEs but also on bacterial chromosomes (6–8). This has raised questions about their roles beyond the maintenance of MGEs (9–12). Although TA systems were linked to bacterial persistence against antibiotics (13–15), this role has recently been called into question (16, 17). The role of chromosomal TA systems remains largely unknown; however, a growing number of data point to them as phage defense elements (18–22).

Staphylococci are gram-positive bacteria that commonly colonize the skin and mucous membranes of humans and animals. The *Staphylococcus* genus comprises 73 species, with the majority considered commensals (23, 24). However, *Staphylococcus aureus* is a notorious opportunistic human pathogen (25). Additionally, *Staphylococcus saprophyticus* frequently causes urinary tract infections, and *Staphylococcus epidermidis* is associated with infections linked to indwelling medical devices. *Staphylococcus haemolyticus*, *Staphylococcus simulans*, *Staphylococcus cohnii*, *Staphylococcus warneri*, and *Staphylococcus lugdunensis* have also been reported as causative agents of infections in humans (26). *Staphylococcus pseudintermedius* is pathogenic to dogs and cats (27).

Staphylococcal pathogenicity is driven by an array of virulence factors, many of which are encoded on MGEs (28). Antibiotic resistance poses a significant threat, particularly in the context of hospital-acquired infections caused by *S. aureus*. Many AR determinants are encoded and disseminated by MGEs (29). For example, pC194 plasmid confers resistance to chloramphenicol (30), and resistance to β-lactam antibiotics is mediated by the SCCmec cassette (31).

The acquisition of MGEs carrying virulence factors and antibiotic resistance genes contributes to the development of highly pathogenic and multidrug-resistant strains, such as *S. aureus* USA300. The latter strain harbors MGEs such as the arginine catabolism mobile element (ACME), Panton-Valentine leukocidin (PVL) toxin, methicillin resistance cassette (SCCmec) as well as plasmids conferring resistance to tetracycline (pUSA02) and erythromycin (pUSA03) (32, 33).

Staphylococci harbor several type II TA systems, which are found on both plasmids and chromosomes. MazEF-Sa is the most widespread, present in nearly all staphylococcal chromosomes, while YefM/YoeB-Sa1 and -Sa2 are also commonly found (34). The Epsilon/Zeta and PemIK-Sa1 TA systems are located on staphylococcal plasmids. PemIK-Sa1 is encoded on the pCH91 (pAvX) plasmid associated with *S. aureus* strains from poultry, which also carries a host-specific virulence factor, the cysteine protease (StpC) (35). Interestingly, PemIK-Sa1 has been identified on a mosaic pPA3 plasmid, which confers erythromycin and cadmium resistance (from pPH2) and tetracycline resistance (from pBC16) (36). PemIK-Sa1 has been shown to stabilize plasmids and regulate the gene expression in bacterial cells. The PemK-Sa1 toxin is a sequence-specific RNase that affects various transcripts without targeting the protein synthesis machinery in staphylococci. Notably, the target sequence (UAUU) is underrepresented in transcripts encoding virulence factors but overrepresented in those for transmembrane transporters (37).

A homologous system, PemIK-Sa1Sp, was identified on the chromosome of *S. pseudintermedius*. In this case, the transfer from plasmid to chromosome resulted in the loss of toxicity of PemK-Sa1Sp, despite retaining its RNase activity (38). PemIK-Sa6, a distant homolog of PemIK-Sa1, was identified within SCCmec cassette of *S. pseudintermedius* AI16, providing yet another example of co-occurrence of PemIK system and methicillin resistance determinants (34). These examples suggest a potential link between TA systems and antibiotic resistance; however, a comprehensive, systematic study is needed to fully elucidate this relationship.

Here, we report the results of a staphylococcal genome analysis aimed at identifying potential correlations between TA systems and antibiotic resistance determinants. Surprisingly, we observed a negative correlation between TA systems and antibiotic resistance. Specifically, our study reveals that the TA system PemIK-Sa6, which co-localizes with SCCmec, is inactive. Moreover, the expression of the PemK-Sa1 toxin is associated with decreased antibiotic resistance in staphylococci. These findings have potential implications for understanding the dynamics of antibiotic resistance in staphylococci.

## RESULTS

### Type II toxin–antitoxin systems correlate negatively with antibiotic resistance determinants

**Fig 1:**
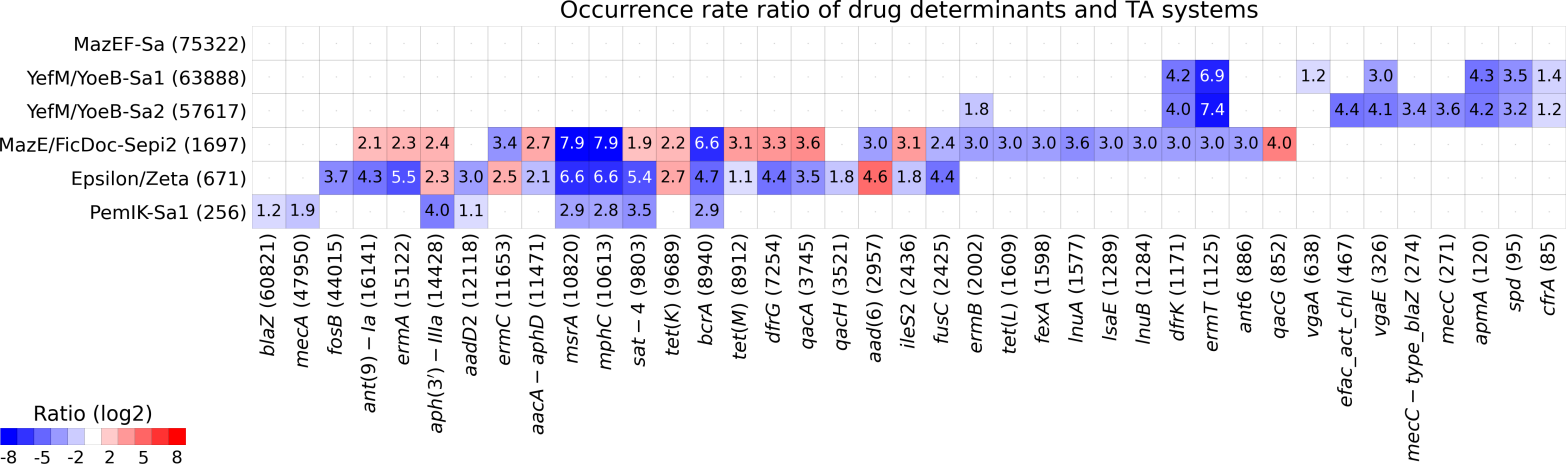
Co-occurrence of antibiotic resistance determinants and TA systems. Ratios are depicted on log2 scale. Values illustrate the rate ratio (how many times higher [red hues] or lower [blue hues]) of drug resistance determinants among strains harboring TA systems compared to all analyzed genomes. Empty fields designate ratios of absolute value below 2.0 (log2 1.0).

To evaluate the potential correlation between TA systems and AR genes in *S. aureus*, we searched for the co-occurrence of both in 75,919 genomes available at the time of analysis (Table S1). The MazEF-Sa was found to be coded in 75,322 genomes (99.21%), followed by YefM/YoeB-Sa1 (63,888; 84.15%), YefM/YoeB-Sa2 (57,617; 75.89%), MazE/FicDoc-Sepi2 (1,697; 2.24%), Epsilon/Zeta (671; 0.88%), and PemIK-Sa1 (256; 0.34%). Regarding AR determinants, we identified 39 genes with *blaZ* (60,821; 80.11%) and *mecA* (47,950; 63.16%) being the most widespread. Other determinants were less prevalent with 0.1% being the lowest recorded value. Next, we correlated the co-occurrence of each TA system with each AR gene (Fig. 1; Fig. S1; Table S2 to S6). Expectedly, the almost complete penetrance of MazEF-Sa was associated with no bias in the co-occurrence of this TA system and any AR genes. In regard to less prevalent paralogous systems YefM/YoeB-Sa1 and YefM/YoeB-Sa2, 7 and 10 AR determinants were biased, respectively, all negatively. The strongest negative correlation of TA system and AR was identified for *ermT*, encoding resistance to macrolide, lincosamide, and streptogramin B (MLS_B_). Only 8 out of 1,125 genomes carrying *ermT* are positive for YefM/YoeB-Sa1, whereas the remaining 1,117 *ermT* genes are distributed within 12,031 YefM/YoeB-Sa1-negative *S. aureus*. This results in *ermT* prevalence of 0.012% in TA-positive strains and 9.284% in TA-negative strains (compared to 1.48% overall prevalence of *ermT*). A strikingly similar bias was observed in the co-occurrence of YefM/YoeB-Sa2 and the *ermT*. Only 5 out of 57,617 TA-positive genomes (0.008% prevalence) and 6.20% (1,120 out of 18,302) TA-negative genomes contained *ermT*. Moreover, 1,117 *ermT*-positive strains (99.28%) were negative for both TA systems. Interestingly, a negative correlation was also identified for *ermB* and YefM/YoeB-Sa2, with a prevalence of 0.76% in TA-positive strains and 8.55% in TA-negative strains.

**Fig 2:**
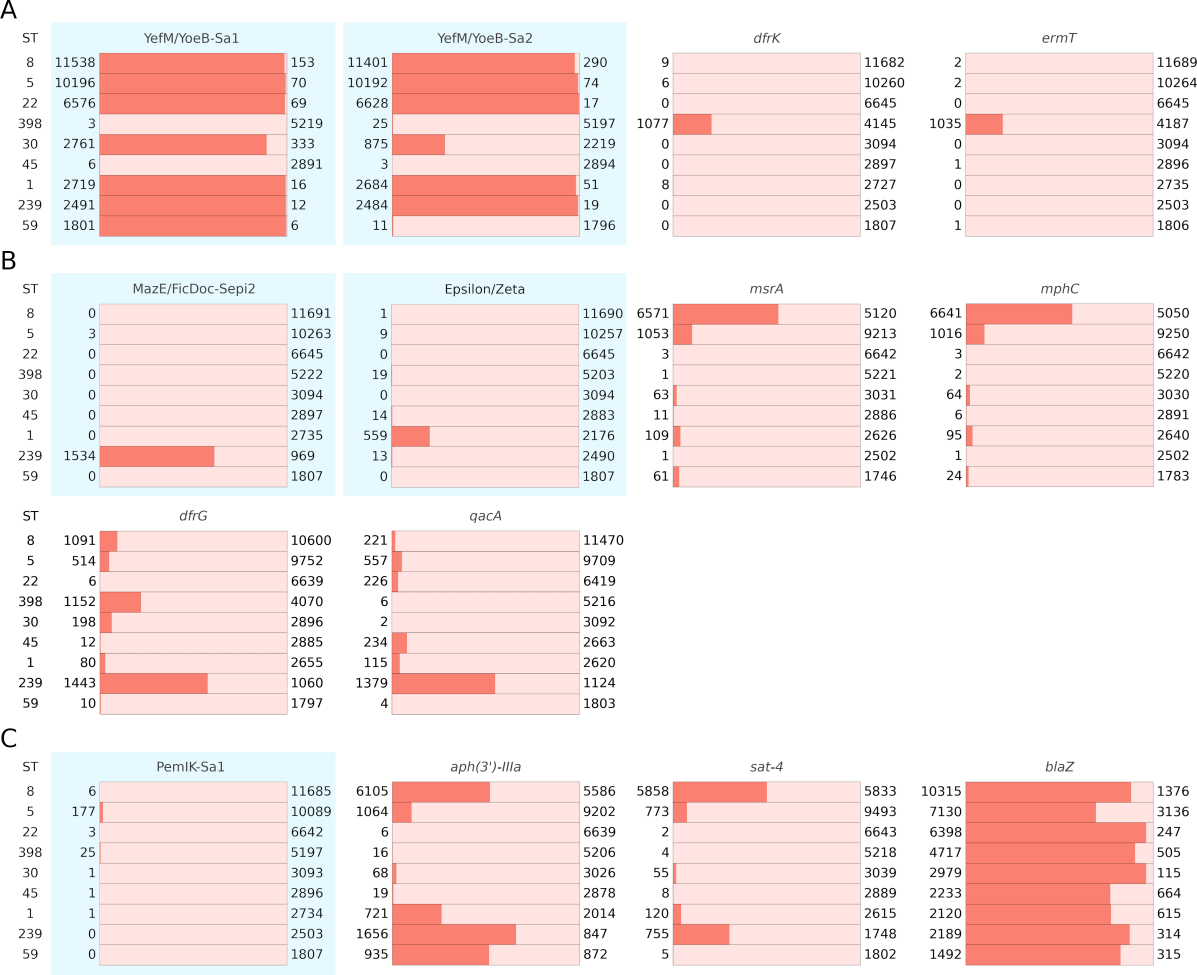
Prevalence of selected TA systems and resistance determinants across the nine most common sequence types (ST) among analyzed genomes. (**A**) YefM-Sa1/YoeB-Sa1 and YefM-Sa2/YoeB-Sa2 are highly prevalent in ST other than 398/45 and 398/45/59, respectively. Resistance determinants *dfrK* and *ermT* are highly frequent in ST398, which is almost devoid of both TA systems. (**B**) TA systems of low prevalence occur largely disjointly with selected resistance determinants, but co-localization of *dfrG* and *qacA* is observed with MazE/FicDoc-Sepi2 and Epsilon/Zeta, respectively. (**C**) For PemIK-Sa1 system, the prevalence of which is very low, a negative co-occurrence with *aph(3')-IIIa* and *sat-4* is observed not only at the genome but also at the ST level. A negative co-occurrence with PemIK-Sa1 system and *blaZ* is observed at the genome level, but not among STs, due to the broad distribution of *blaZ*. Charts referring to TA systems are highlighted. Numbers indicate the number of strains positive/negative for a respective determinant in a respective ST.

*In silico* multilocus sequence typing (MLST) analysis further strengthens the above-described negative correlations. YefM/YoeB-Sa1 and YefM/YoeB-Sa2 are almost non-existent in sequence type (ST) 398 and 45, and YefM/YoeB-Sa2 is rare in ST59. At the same time, *ermT* and *dfrK* are almost exclusively present in ST398, whereas *ermB* is present in ST59 (Fig. 2A; Fig. S1 and S2).

MazE/FicDoc-Sepi2 distribution is also biased in regard to co-occurrence with 26 AR determinants; however, the bias is bidirectional in this case. For 15 AR determinants, the correlation was negative, whereas for the remaining 11 AR determinants, it was positive. The most negative correlation in the distribution of MazE/FicDoc-Sepi2 and AR was identified for *msrA*, conferring erythromycin and type B streptogramins resistance (39, 40), and for *mphC*, encoding resistance to erythromycin and spiramycin (41). Specifically, single *msrA* and *mphC* genes were found in 1,697 MazE/FicDoc-Sepi2-positive genomes (prevalence: 0.059%), whereas the remaining 10,820 and 10,613 were present in TA-negative strains (prevalence: 14.58% and 14.30%), respectively. This perfectly fits the prevalence of MazE/FicDoc-Sepi2, as well as *msrA* and *mphC* prevalence in ST types (Fig. 2B).

The strongest positive correlations were observed for *dfrG* (trimethoprim resistance [42, 43]) and *qacA* (44, 45), mediating resistance to a wide array of monovalent or divalent cationic, lipophilic, and antimicrobial compounds. The *dfrG* gene was identified in 1,560 out of 1,697 MazE/FicDoc-Sepi2-positive strains (91.93% prevalence), whereas the remaining 5,694 *dfrG* genes were distributed within 77,222 TA-negative *S. aureus* strains (7.67% prevalence). The prevalence of *qacA* followed an identical trend, with 59.87% prevalence among MazE/FicDoc-Sepi2-positive strains and 3.68% prevalence among TA-negative strains. Moreover, the prevalence of *aph(3’)-IIIa* (resistance to aminoglycosides) within MazE/FicDoc-Sepi2-positive strains was strikingly high (98.64%; 1,647 out of 1,697 strains). Interestingly, both determinants co-localized on the same contig in 87.8% of the TA-positive genomes, indicating close genomic localization (Fig. S3; Table S6).

We identified a biased distribution of 19 AR determinants with respect to the Epsilon/Zeta TA system, with mostly negative correlations observed. Only the presence of *aad*(6) (conferring resistance to aminoglycosides including streptomycin), *tet(K)* (resistance to tetracycline), *ermC* (resistance to erythromycin), and *aph(3’)-IIIa* (resistance to aminoglycosides) correlated positively with Epsilon/Zeta TA system. The penetrance in Epsilon/Zeta-positive strains was high (from 83.8% to 95.5%), whereas in TA-negative strains, it was 5 to 30 times lower. Additionally, *aad(6*) and *aph(3’)-IIIa* were identified together with operons coding for Epsilon/Zeta in 639 contigs [among 640 total strains positive for *aad(6*), *aph(3’)-IIIa*, and Epsilon/Zeta] of relatively low length (below 10 kb), which suggests genetic coupling of the AR and TA determinants (Fig. S4; Table S6). The correlation between the presence of Epsilon/Zeta and the remaining 15 AR genes was negative. The most pronounced negative correlation was observed for *msrA*, *mphC,* and *ermA*, all conferring resistance to erythromycin.

The plasmid-encoded PemIK-Sa1 system was the least widespread among the TA systems analyzed in this study (256 of 75,919 analyzed strains) and correlated exclusively negatively with eight AR determinants. The genes *aph(3’)-IIIa* and *sat-4* (both conferring nucleoside resistance) were the least prevalent among PemIK-Sa1-positive strains (1.17% compared to 19.00% and 12.91% prevalence expected for random distribution, for *aph(3’)-IIIa* and *sat-4*, respectively). β-lactam resistance determinants *blaZ* and *mecA* are widespread in *S. aureus* genomes, but their prevalence in PemIK-Sa1-positive strains (35.55% and 17.19%, respectively) is markedly lower than in TA-negative strains (80.26% and 63.32%, respectively). In line with the above findings, PemIK-Sa1 is almost exclusively found in ST5, where the prevalence of *aph(3’)-IIIa*, *sat-4*, and *blaZ* is among the lowest in this genetic background (Fig. 2C). In summary, we found that the vast majority of type II TA systems in *S. aureus* negatively correlate with AR genes with only a single exception of MazEF-Sa. The trend is observed for both high-prevalence TA systems (YefM/YoeB-Sa1 and Sa2) and low-prevalence ones (MazE/FicDoc-Sepi, Epsilon/Zeta, and PemIK-Sa1).

### Putative PemK-Sa6 toxin is neither toxic nor functional

In this study, we demonstrated a negative correlation between PemIK-Sa1 and methicillin resistance determinants in *S. aureus*. Recently, however, we identified PemIK-Sa6, a distant homolog of PemIK-Sa1, encoded within the SCCmec_AI16_ of *S. pseudintermedius* AI16 (34). To address the apparent contradiction regarding the exclusive nature of PemIK TA systems and methicillin resistance, we investigate PemIK-Sa6 in more detail here.

**Fig 3:**
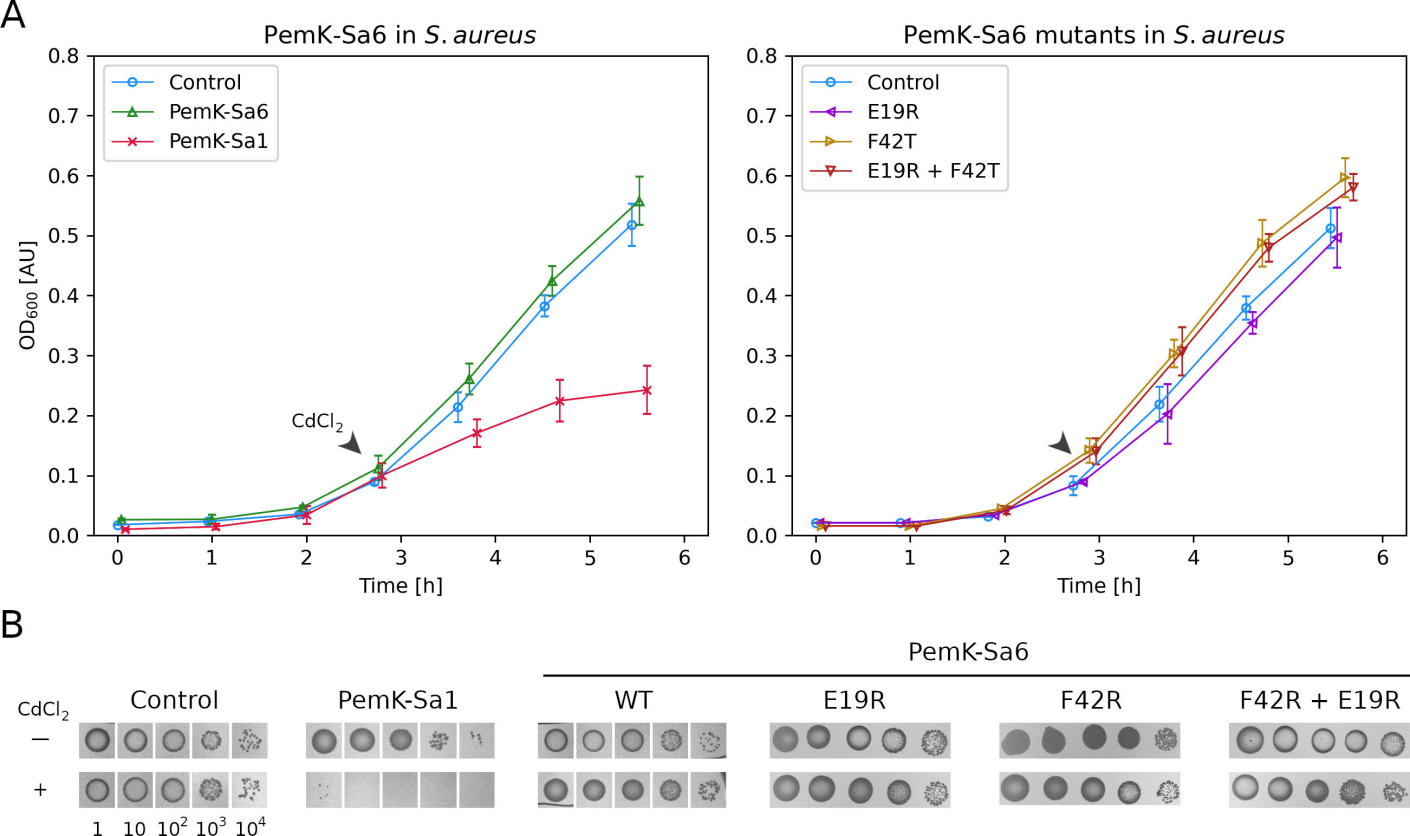
Impact of PemK-Sa6 and its mutants on the growth of *S. aureus*. (**A**) Cadmium-induced expression of PemK-Sa1 but not PemK-Sa6 inhibits the growth in liquid culture (left chart). E19R and F42T mutations at the catalytic site of PemK-Sa6 do-not-restore toxicity (right chart). The arrow indicates CdCl_2_-mediated induction of toxin expression. (**B**) Similar results are observed on solid media.

The PemK-Sa6 toxin coding sequence was cloned into an expression plasmid under the control of an inducible promoter, and the anticipated toxicity of the gene product was determined in *Escherichia coli*. Induction of toxin expression with isopropyl β-d-1-thiogalactopyranoside (IPTG) had no effect on growth in liquid or solid media (Fig. S5), suggesting the PemK-Sa6 toxin is inactive. Although *E. coli* toxicity assay is routinely used to evaluate the toxicity of TA system-encoded toxins from diverse species, we could not have excluded a *Staphylococcus*-specific target of PemK-Sa6. To test this possibility, *pemK-Sa6* was cloned into pCN51 shuttle vector and expressed in *S. aureus*. No effect on growth dynamics was observed. Because we were using *S. aureus*, and not *S. pseudintermedius* AI16 where PemIK-Sa6 was originally identified, we additionally tested a variant where the native ribosome-binding site was exchanged to that of *pemK-Sa1* to ensure comparable expression to PemK-Sa1, which serves as a positive control for toxin toxicity. No effect on growth dynamics was observed (Fig. 3; Fig. S6). However, to exclude even minor differences in the expression of *pemK-Sa6* within *Staphylococcus* genus, we transformed *S. pseudintermedius* LMG2221 with pCN51*-pemK-Sa6* or *-pemK-Sa1*. Again, no growth inhibition was observed for the strain expressing PemK-Sa6 (Fig. S6C).

**Fig 4:**
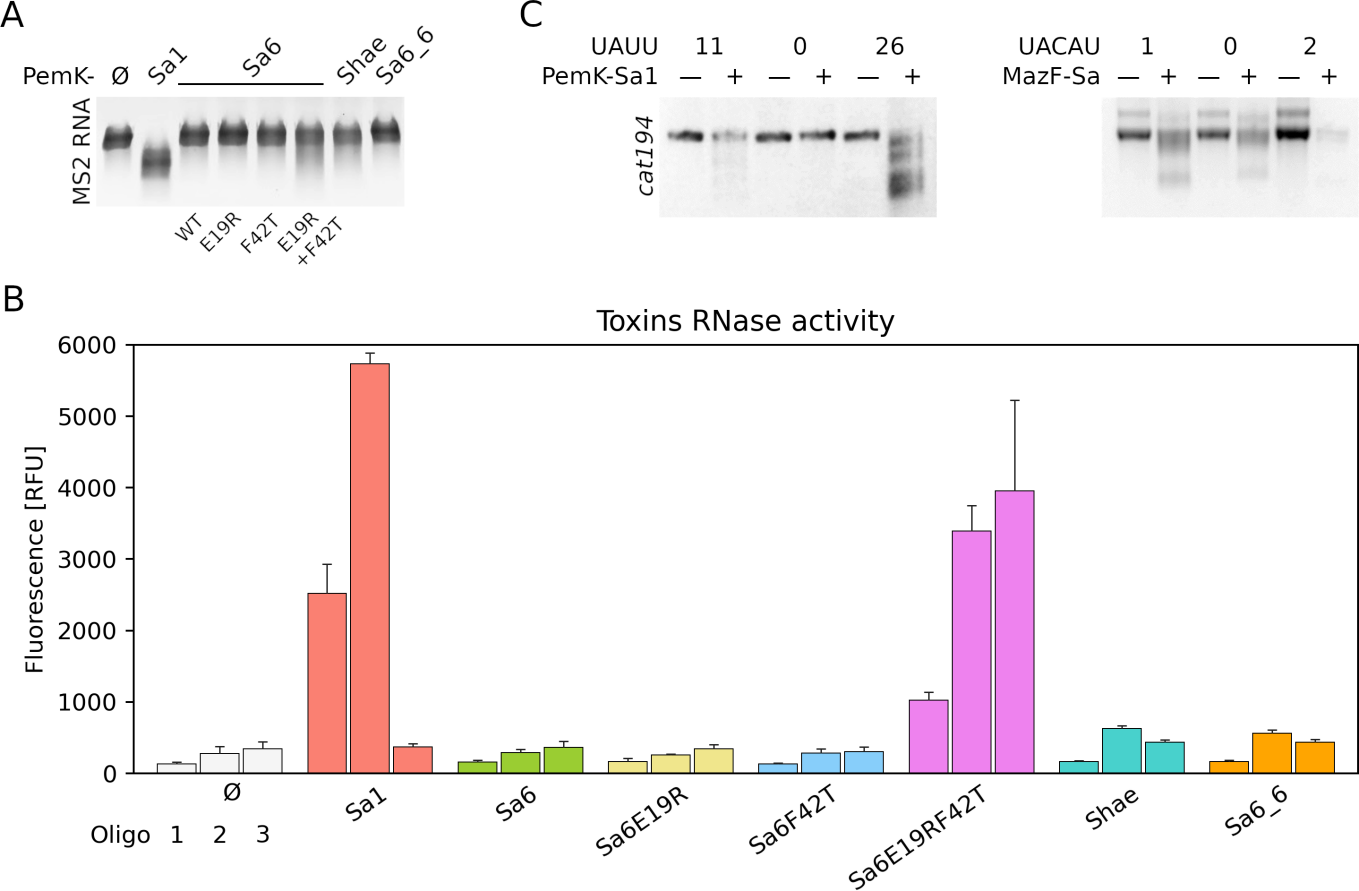
Assessment of RNase activity of MazF-Sa, PemK-Sa1, PemK-Sa6, and mutants against different substrates. (**A**) PemK-Sa6 and its E19R and F42T mutants do not exhibit detectable activity against phage MS2 RNA. Very low activity is observed for the double mutant. PemK-Sa1 displays a strong RNase activity. (**B**) RNase activity of PemK-Sa6 toxin and its mutants against fluorescently labeled oligo RNAs. PemK-Sa6 single mutants as well as PemK-Shae are enzymatically inactive. Double-mutant PemK-Sa6 (E19R + F42T) shows activity comparable to PemK-Sa1. (**C**) RNase activity of PemK-Sa1 and MazF-Sa against *cat194* transcripts containing indicated numbers of target sequences (UAUU and UACAU, respectively). MazF-Sa displays residual unspecific activity visible as digestion of transcripts devoid of target UACAU sites.

**Fig 5:**
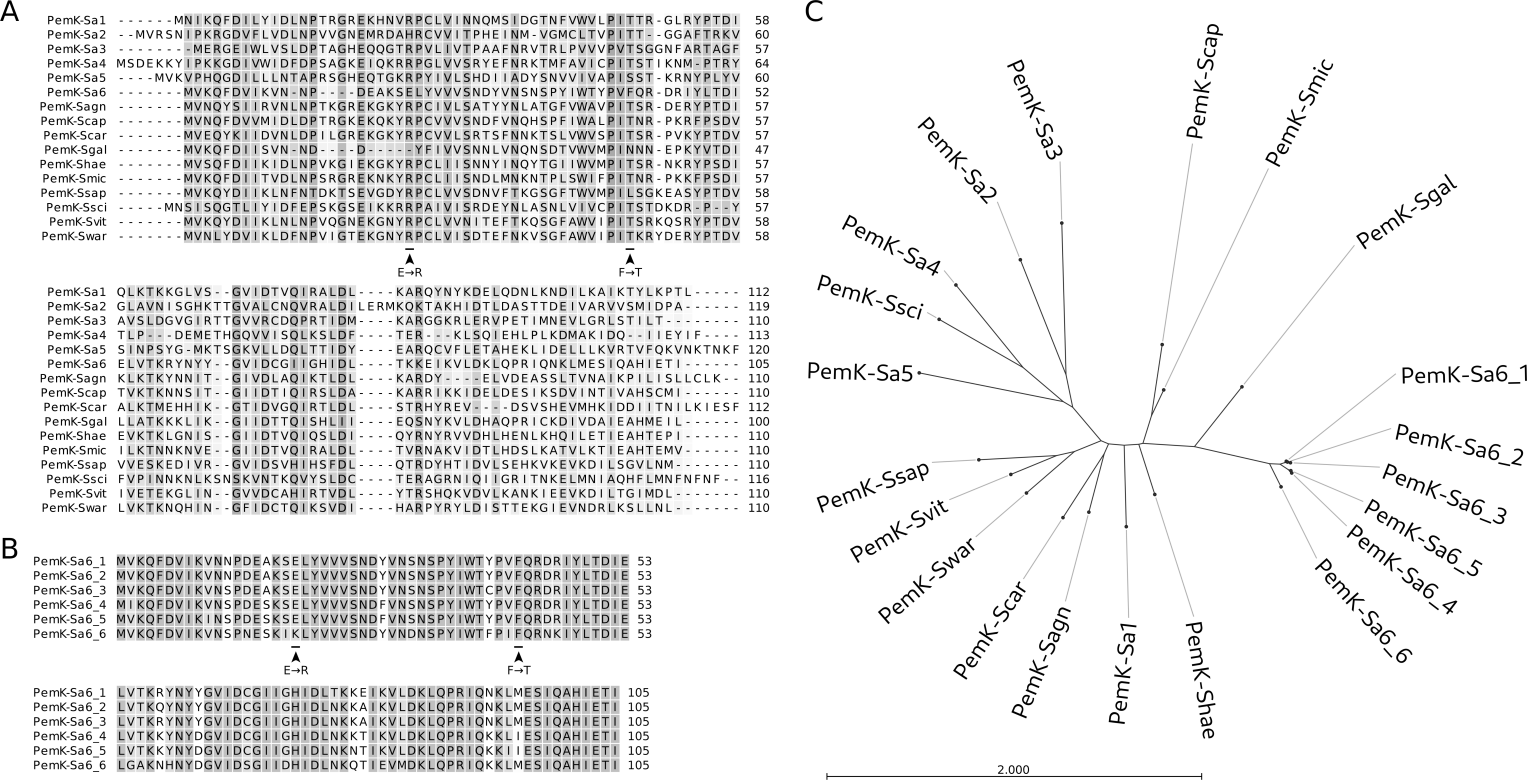
Phylogenetic relations among PemK-Sa6 variants and other staphylococcal PemK homologs. (**A**) Similarity among all representative sequences of staphylococcal PemK family toxins. Mutation sites in PemK-Sa6 are indicated under the multiple sequence alignment. These are two insertions and two point mutations making the sequence more similar to the active PemK-Sa1. (**B**) Similarity among all unique sequences of PemK-Sa6 homologs with the aforementioned mutations indicated. (**C**) Phylogenetic tree of representative sequences of staphylococcal PemK family toxins and all unique sequences of PemK-Sa6 homologs. PemK-Sa6_1 is synonymous to PemK-Sa6, the toxin that belongs to PemIK-Sa6 system initially discovered in the SCCmec_AI16_ element of *S. pseudintermedius* AI16. Among all homologs of this toxin, PemK-Sa6_6 is the most distant and simultaneously the most similar to PemK-Sa1, which has been characterized as enzymatically active. PemK-Shae is closest to PemK-Sa1 toxin but remains uncharacterized.

The experiments described in the prior paragraph suggest that PemK-Sa6 does not have a global toxic effect. The toxin may have a more subtle effect, as shown earlier for PemK-Sa1Sp from *S. pseudintermedius*. Overexpression of PemK-Sa1Sp has not influenced the growth, but the protein exhibited an evident RNase activity (38). We expressed and purified the recombinant PemK-Sa6 and tested its effect on MS2 phage RNA. No degradation was observed, which suggests PemK-Sa6 is inactive as RNAse, explaining the lack of toxicity (Fig. 4A). Sequence alignment of PemK-Sa6 and PemK-Sa1 (toxic RNase) and PemK-Sa1Sp (non-toxic RNase) revealed that residues at the catalytic site of PemK-Sa6 are noncanonical (Fig. 5A; Fig. S10). We replaced these residues with those found in catalytically active PemK toxins (E19R, F42T, and a double mutant) and verified the toxicity as well as RNase activity of the mutants. Neither tested variant was toxic to *E. coli* and *S. aureus* when ectopically expressed (Fig. 3; Fig. S5 and S6). Both E19R and F42T variants were inactive in MS2 RNA digestion assay. Only the double mutant was characterized by minor activity compared to negative control, but the activity was negligible compared to that of PemK-Sa1. To further test the RNase activity of PemK-Sa6, short fluorogenic substrates containing target sequences of PemK-Sa1 (oligo1 and oligo2) and MazF-Sa (oligo3) toxins were tested. PemK-Sa1 efficiently hydrolyzed oligo1 and oligo2 but not oligo3. PemK-Sa6 and E19R and F42T single amino acid substitutions were inactive. The E19R, F42T double mutant hydrolyzed all three tested substrates, with the highest activity toward oligo3 (Fig. 4B).

Overall, the above results suggest that PemK-Sa6 has lost its RNase activity and, thus, its toxic effect. Hence, the PemIK-Sa6 TA system is non-functional and unlikely to be involved in the maintenance of SSCmec. These findings support the negative correlation between PemIK TA systems and methicillin resistance: the genetic transfer of PemIK into SCCmec may lead to inactivation of the TA system.

### Chromosomally encoded homologs of PemK-Sa6 are also inactive

In light of the data presented in the prior paragraph, we asked whether chromosomally encoded PemK-Sa6 homologs are enzymatically active. This question was further motivated by the fact that plasmid-encoded PemK-Sa1 is highly toxic, whereas its homolog (PemK-Sa1Sp), encoded within the chromosome of all *S. pseudintermedius* strains, is non-toxic but enzymatically active (38). We selected PemK-Sa6_6 and PemK-Shae because, in the multiple sequence alignment phylogeny tree, the former branches closely to PemK-Sa6 while the latter branches closely to PemK-Sa1 (Fig. 5). Coding sequences for both toxins were cloned into expression vectors, and the respective proteins were purified and tested for enzymatic activity using RNA MS2 and synthetic fluorogenic substrates described earlier in this study. Neither of the proteins exhibited RNase activity (Fig. 4A and B). These findings strengthen our earlier observation that plasmid-to-chromosome transfers of PemIK TA systems are related to decreased or lost toxicity of the toxic component of the system.

### Expression of PemK-Sa1 decreases resistance to chloramphenicol

The negative correlations of TA and AR determinants identified in this study suggest functional influence. We hypothesized that TA systems have negative effect on AR, thus exert a strong evolutional pressure to select against TA systems upon environmental exposure to antibiotics. To test this hypothesis, we chose MazEF-Sa and PemIK-Sa1, two well-characterized and functionally related TA systems, and evaluated their potential influence on antibiotic resistance in *S. aureus*. MazEF-Sa is chromosomally encoded in virtually all *S. aureus* strains. PemIK-Sa1 is carried on plasmids and is found in 0.34% of strains. Both PemK-Sa1 and MazF-Sa toxins are sequence-specific RNases specific toward UAUU and UACAU, respectively.

*S. aureus* was transformed with a plasmid carrying resistance to chloramphenicol and expressing tested toxins from cadmium-inducible promoter. Minimal inhibitory concentration (MIC) values for the antibiotic were determined in the presence and absence of cadmium induction. The addition of the inducer has no influence on MIC in bacteria transformed with the control plasmid. However, we observed a 2.5-fold decrease in MIC upon PemK-Sa1 expression, while the expression of MazF-Sa had no effect (Table 1). To elucidate if the decrease in MIC was driven by RNase activity against *cat194* transcript, two variants of the plasmid were prepared. In the first, the PemK-Sa1 target sequences were removed from *cat194*, whereas in the second, we introduced the maximal possible number (26) of target (UAUU) sequence, without altering the sequence of Cat194 chloramphenicol acetyltransferase. Upon chloramphenicol challenge, the UAUU-null variant exhibited only slightly decreased MIC (60 vs 80 µg/mL), whereas in the UAUU-max variant, a threefold decrease in MIC was observed, compared to the control (20 vs 60 µg/mL). Interestingly, despite the identical protein sequence of Cat194, the coding sequence seems to influence its expression/activity, because *S. aureus* strains carrying the modified variants of *cat194* (and no TA system) exhibited lower MIC compared to a strain expressing the native *cat194* gene (60 and 80 vs 100 µg/mL). When an analogous experiment was performed with *cat194* optimized for MazF-Sa target sites (0, 1, or 2 UACAU sites), no differences in MIC were observed.

**TABLE 1:** Minimal inhibitory concentration (MIC) of chloramphenicol for *S. aureus* carrying *cat194* and toxin genes^*a*^

Toxin	CdCl_2_	MIC for chloramphenicol (µg/mL)					
		PemK-Sa1 (no. of UAUU)			MazF-Sa (no. of UACAU)		
		(13; WT)	(26; max)	(0; none)	(1; WT)	(2; max)	(0; none)
–	–	100	60	80	100	100	100
–	+	100	60	80	100	100	100
+	–	100	60	80	100	100	100
+	+	40	20	60	100	100	100

^*a*^ Cadmium ions induce toxin expression.

To further evaluate the susceptibility of *cat194* transcripts to PemK-Sa1, we put together the respective transcripts with PemK-Sa1 toxin. As expected, the toxin efficiently digested the transcripts containing UAUU, whereas the null variant was barely affected (Fig. 4C).

**Fig 6:**
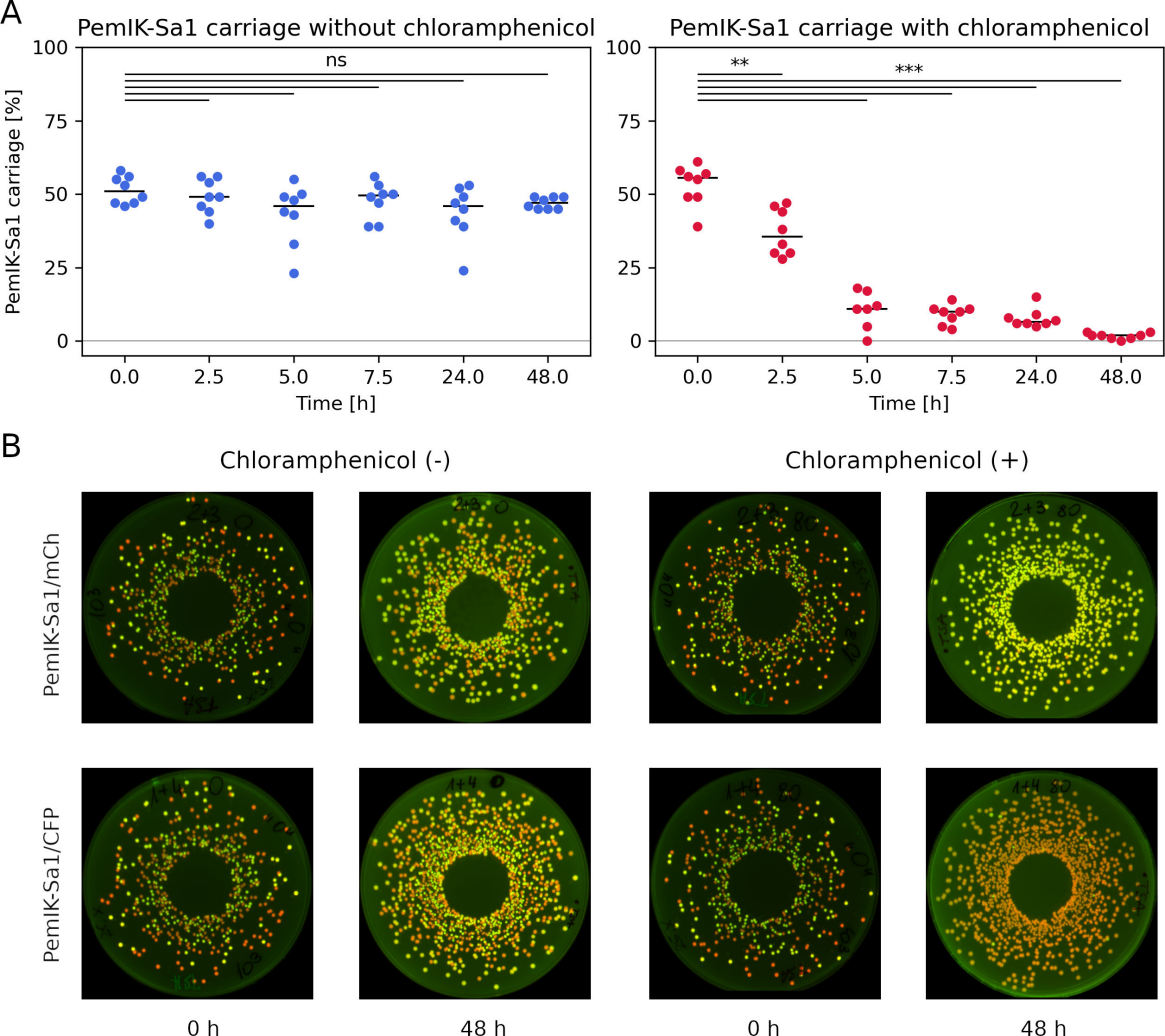
Negative selection between PemIK-Sa1 and *cat194* chloramphenicol resistance determinant. (**A**) In a medium without antibiotic co-cultures of chloramphenicol-resistant *S. aureus* strains, the carriage of PemIK-Sa1 remains constant and oscillates around an initial 50%. However, when chloramphenicol is introduced to the environment, the PemIK-Sa1 carrier is rapidly outcompeted by the control strain (***, *P* ≤ 0.001; **, *P* ≤ 0.010). (**B**) Exemplary spiral plater results for the analyzed co-cultures in the absence and presence of chloramphenicol. The PemIK-Sa1 carrier expresses mCherry (red colonies) and the control strain CFP (green colonies) (upper row), the opposite setting (lower row).

Induced expression of PemK-Sa1 toxin may overestimate its effect and neglect the role of the antitoxin. Therefore, we transformed *S. aureus* with a plasmid encoding an intact PemIK-Sa1 system under the control of its native promoter or a control plasmid. Transformants carrying PemIK-Sa1 exhibited lower resistance to chloramphenicol (MIC 120 µg/mL) than those with the control plasmid (170 µg/mL). Next, we mixed strains with and without the PemIK-Sa1 system in equal proportions and cultured them in a medium with or without the antibiotic. The culture grown without antibiotic pressure retained the initial proportion of the bacteria throughout the experiment (48 h). Strikingly, in the presence of a subinhibitory concentration of chloramphenicol (80 µg/mL), the culture was gradually dominated by the bacteria without the PemIK-Sa1 TA system (Fig. 6). This indicates that the TA system, in the environment containing the antibiotic, is a burden for bacteria, impairing their competition with the bacteria without the system.

In summary, we demonstrated that the expression of PemK-Sa1 decreases the resistance of *S. aureus* to chloramphenicol mainly through the degradation of the *cat194* transcript, whereas the expression of MazF-Sa does not affect the resistance. Moreover, the bacteria carrying the intact PemIK-Sa1 TA system in the presence of the antibiotic are quickly outcompeted by those without the system. There is, therefore, a strong selective pressure on the segregation of PemIK TA systems and antibiotic resistance or inactivation of the system in antibiotic-resistant strains.

## DISCUSSION

*S. aureus* is a major pathogen of humans and animals; however, other staphylococci have recently been considered a serious threat. The major concern from a clinical point of view is the growing antibiotic resistance among staphylococci (46–48). AR is mainly spread by horizontal gene transfer of MGEs, such as plasmids and transposons (49). TA systems are widespread in bacteria, including staphylococci (50, 51), and maintenance of MGEs has been traditionally attributed to TA systems. Toxins of TA systems have even been proposed as targets and/or tools to combat antibiotic-resistant bacteria (52–54). We previously identified and characterized the PemIK-Sa1 TA system. Apart from plasmid maintenance, the system plays a regulatory role in gene expression related to its RNase activity (37). We further identified the PemIK-Sa1 system on a mosaic pPA3 plasmid, which confers resistance to erythromycin and tetracycline (36). As such, one could link the PemIK-Sa1 with the maintenance and spread of antibiotic resistance in *S. aureus*. Furthermore, the chromosomally encoded MazEF-Sa was previously linked to β-lactam antibiotic susceptibility/resistance, as its deletion resulted in increased susceptibility to penicillin (55). The link between TA and AR is, however, based on circumstantial evidence, and broader studies are missing.

This study provides a global overview of the co-occurrence of type II TA systems and AR determinants in *S. aureus*. The most widespread MazEF-Sa system is characterized by virtually complete penetrance and is, therefore, not expected to correlate with AR genes. We find, however, that all known type II TA systems in *S. aureus* other than MazEF-Sa correlate strongly and mostly negatively with a number of AR genes. This finding is unexpected, considering the earlier stipulated maintenance role of TA systems for MGEs given that the spread of AR is largely mediated by MGEs. Such consistent and strong negative correlation may suggest functional links. We suspect that activation of TA systems alters resistance to certain antibiotics, exerting negative selection pressure on the strains harboring both TA systems and AR genes.

YoeB toxins of paralogous YefM/YoeB-Sa1 and YefM/YoeB-Sa2 TA systems of *S. aureus* are ribosome-dependent RNases that inhibit translation initiation (56). Prior work suggested a high prevalence of YefM/YoeB systems as members were identified in 77 of 78 clinical isolates resistant to methicillin methicillin-resistant *S. aureus* (MRSA) (57). Our large-scale analysis indicates that the overall prevalence of YefM/YoeB-Sa1 and Sa2 in MRSA is lower (65.1% and 68.5%, respectively). In addition, we observed no bias in distribution among MRSA (*mecA*+) and methicillin-susceptible *S. aureus* (MSSA) (*mecA*-) strains (Table S3). An earlier study found no difference in the MIC for cefazolin and vancomycin between wild-type and *yoeB* mutants. In turn, the loss of *yoeB-Sa1* and *yoeB-Sa2* resulted in a slight, but significant, decrease in the rate of persister cell formation (58). Moreover, both *yefM/yoeB-Sa1* and *yefM/yoeB-Sa2* transcripts were upregulated upon exposure to subinhibitory concentrations of erythromycin and tetracycline but not to vancomycin (59). Erythromycin is a macrolide antibiotic that acts by inhibiting protein synthesis by binding to the 23S ribosomal RNA molecule in the 50S subunit of ribosomes. Resistance to erythromycin is conferred either by *erm* genes, encoding methyltransferases that modify 23S rRNA, thus reducing the macrolide ability for ribosome binding, or by *msr* genes, which encode efflux pumps (60). Taking all these into account, it is tempting to speculate that the negative correlation between some *erm* (*ermT* and *ermB*) genes and YefM/YoeB-Sa1 and YefM/YoeB-Sa2 is related to activation of these TA systems by the exposure to erythromycin. According to that hypothesis, while erythromycin resistance protects from the direct action of the antibiotic, the activation of the TA system by the antibiotic results in bacteriostasis that still overrides the advantage of the resistance gene.

Epsilon-Zeta family TA systems were originally identified in a broad-host-range and multidrug-resistance-conferring pSM19035 staphylococcal plasmid. TA systems of this family have also been reported in other bacteria and encompass a number of more recently studied PezA/PezT system homologs (61–63). Although operons coding for Epsilon/Zeta systems are found primarily in plasmids, chromosomal location has also been reported in certain species (63). Zeta toxins are kinases that disrupt cell wall synthesis by targeting UDP-N-acetylglucosamine (UNAG), a crucial peptidoglycan precursor (64). Due to low prevalence, we were able to identify only a single earlier report of *epsilon/zeta* in a staphylococcal genome of a *cfrA*-positive MRSA strain CM05 (65). In that study, the authors described a chromosomal insertion of a sequence similar to pSM19035 fragment into 23S rRNA allele 4. The inserted fragment harbored several methyltransferase genes conferring resistance to aminoglycosides (e.g., erythromycin) and an *epsilon/zeta* locus. This earlier case finding aligns with our results demonstrating chromosomal location of *epsilon/zeta* loci in *S. aureus*, and explains positive co-occurrence and close co-localization of o *epsilon/zeta* operons with aminoglycosides resistance determinants *aph(3')-IIIa* and *aad*(6) (Table S6). Chromosomal localization of *epsilon/zeta* operons also explains the positive co-occurrence with chromosomally encoded *ermC* and *tet(K*). Considering that staphylococcal Epsilon/Zeta TA system has not been characterized to any extent, it is difficult to speculate how the presence of this TA system may affect resistance conferred by AR determinants for which a clear negative correlation with the TA system was identified in this study.

All biased AR determinants correlated negatively with PemIK-Sa1, similar to YefM/YoeB TA systems. PemIK-Sa1 system was also the only identified TA system characterized by (negatively) biased co-occurrence with determinants conferring resistance to β-lactams (*blaZ* and *mecA*), which are highly prevalent in *S. aureus*. PemIK-Sa1 is a TA system harboring highly toxic PemK-Sa1 toxin with RNase activity (37). Although the natural trigger that releases the toxin remains unknown, it is clear that the release of PemK-Sa1 activity generates a high burden for a cell. Such negative influence must be compensated by other benefits to allow stable maintenance of the system in *S. aureus*. PemIK-Sa1 is encoded on plasmids, which additionally carry the gene coding for staphopain C, a cysteine protease, which is a poultry-host-specific virulence factor (66). Indeed, a vast majority of PemIK-Sa1-positive strains identified in this study are of avian origin. This would suggest that the compensation of the PemK-Sa1-related burden is provided through maintenance of genetic elements carrying an important virulence factor. Interestingly, the prevalence of *mecA* in TA-positive strains from poultry is lower (5.98%) compared to the average (17.19%), pointing to a stronger selection pressure for maintenance of PemIK-Sa1-encoding plasmids than SCCmec antibiotic resistance in these hosts.

All TA systems with exclusively negative correlation with AR determinants identified in this study in *S. aureus* encompass toxins with RNase activity. We hypothesized that the negative correlation may occur because the toxins could affect the stability of AR gene transcripts. Homologous RNase toxins have been identified together with AR determinants in species other than *S. aureus*, for example, *pemIK-Sa6*, which is localized within the SCCmec_AI16_ of *S. pseudintermedius* AI16. This apparent contradiction is resolved when RNase activity is analyzed, because mutations in the toxin gene result in loss of RNase activity in PemK-Sa6. Using mutagenesis, we were able to restore the activity, but the mutein was still not toxic, suggesting an accumulation of functional mutations outside the active site. Furthermore, in contrast to *S. aureus*, all strains of *S. pseudintermedius* carry the chromosomally encoded PemIK-Sa1Sp system, and *mecA*-driven resistance to β-lactams is widespread in these bacteria (methicillin-resistant *S. pseudintermedius*, MRSP). In this case, PemK-Sa1Sp is an active RNase but has no toxic effect (38). It is tempting to speculate that the elimination of the toxic effect/activity of PemK eliminates its negative pressure on the maintenance of SCCmec. To further verify this assumption, we tested PemK-Sa6_6 and PemK-Shae, both encoded on staphylococcal chromosomes. Neither protein exhibited detectable RNase activity or toxicity. The lack of activity of PemK-Sa6_6 is explained by T42F mutation. In PemK-Shae, the catalytic residues are identical to toxic PemK-Sa1; hence, other cumulated mutations must be responsible for the loss of RNase activity (Fig. 5A). The above examples suggest that chromosomal transfer of PemIK TA systems is associated with loss of toxicity and often associated with loss of RNase activity.

It has been demonstrated earlier that the toxicity of PemK-Sa1 is related to its RNase activity (37). Here, we experimentally verified the toxin’s influence on resistance to antibiotics, exemplified by chloramphenicol. We tested MazF-Sa in parallel because the protein, like PemK-Sa1, is a sequence-specific RNase, but unlike PemK-Sa1, MazF-Sa is encoded in all *S. aureus* strains and hence does not correlate with AR. The *cat194* is a plasmid-harbored, directly regulated gene encoding acetyltransferase conferring resistance to chloramphenicol (30). Our results clearly show that the expression of PemK-Sa1 increases the susceptibility of chloramphenicol-resistant *S. aureus* strains. The phenomenon is linked to the number of PemK-Sa1 target sites (UAUU) in the *cat194* gene transcripts. UAUU-null transcripts were not affected by PemK-Sa1, whereas those containing target sequences were efficiently degraded. However, the direct degradation of the *cat194* transcript is only partially responsible for the decreased resistance to chloramphenicol in PemK-Sa1-positive strains. We demonstrated that a strain carrying TATT-null variant of *cat194* still exhibits slightly increased susceptibility to chloramphenicol when PemK-Sa1 is expressed. Because the target sequence of PemK-Sa1 is prevalent in the staphylococcal transcriptome, degradation of transcripts of other genes may have an indirect effect on resistance.

We suspected that mechanisms similar to those summarized in the prior paragraph may be responsible for negative correlations observed between other TA systems and other AR genes. All AR genes analyzed in the study contain a number of the toxin target sequences, rendering their transcripts susceptible to degradation (Table S7). However, there is no significant difference in the number and the frequency of UAUU in gene transcripts of AR determinants co-occurring and non-occurring with PemIK-Sa1 (Fig. S7 to S9). Interestingly, UAUU is overrepresented in transcripts for genes encoding transmembrane transporters, which often serve as efflux pumps (37), and decreased antibiotic concentration within bacterial cells by active efflux is one of the mechanisms of the resistance. In consequence, *S. aureus* strains carrying both PemIK-Sa1 TA system and AR determinants would be characterized by decreased antibiotic resistance, compared to TA-negative strains. Such a mechanism would exert negative selective pressure on AR-positive and PemIK-Sa1-positive strains in the presence of antibiotics (Fig. 6). As an evolutionary consequence, the loss of TA systems would be expected, which could explain the negative correlation in the co-occurrence of TA and AR observed in this study. Another mechanism could involve the detoxification of the toxin, as demonstrated in this study in the case of SCCmec and chromosomally encoded PemK-Sa1 homologs.

In conclusion, although TA systems stabilize MGEs, which may spread AR determinants as cargo, a number of negative correlations between the co-occurrence of type II TAs and ARs have been identified in *S. aureus*. Especially in the case of RNase toxins, an exclusively negative bias was observed. We demonstrated a mechanism responsible for this negative correlation where the toxin negatively affects the level of antibiotic resistance by degrading the resistance gene transcripts. We also demonstrated that evolutionary pressure results in inactivation/detoxification of chromosomally encoded TA systems. Considering the limitations of this study, we have to point out that our claims are supported by experimental evidence that is limited to a single TA system (PemIK-Sa1 and its homologs) and a single antibiotic determinant (exemplified by chloramphenicol) in a single bacterial species (*S. aureus*).

## MATERIALS AND METHODS

### Bacterial strains and growth conditions

Unless otherwise specified, *E. coli* and *Staphylococcus* spp. strains were grown at 37°C in Luria-Bertan (LB) Agar and LB Broth (Difco) or in Tryptone Soya Agar and Tryptone Soya Broth (Oxoid), respectively. Liquid cultures were agitated at 180 rpm. Transformed *E. coli*, *S. aureus*, and *S. pseudintermedius* cultures were supplemented with ampicillin (100 µg/mL), chloramphenicol (10 µg/mL, unless otherwise specified), and erythromycin (10 µg/mL), respectively. *E. coli* BL21 (DE3) Gold (Agilent) strain was used for protein expression as well as growth inhibition assays alongside *S. aureus* RN4220 (67) and *S. pseudintermedius* LMG22221. *S. pseudintermedius* LMG22221 and AI16 (68) were obtained from The Belgian Coordinated Collections of Microorganisms and provided by the courtesy of Prof. Vincent Perreten, respectively.

### Plasmid constructs used in the study

For expression in *E. coli*, constructs based on pETDuet-1 plasmid (Novagen) were used. Expression in *S. aureus* and *S. pseudintermedius* was achieved from constructs based on pCN51 plasmid with cadmium-inducible promoter, a promoter-less pCN35 plasmid (69), and pALCP2G plasmid with a promoter from PemIK-Sa1 TA system (37). The constructs were prepared by restriction digestion and ligation as described elsewhere (70), and were further mutated using QuikChange Site-Directed Mutagenesis Kit (Agilent). PemK-Sa6 coding sequence was cloned from *S. pseudintermedius* AI16. PemK-Sa6_6 and PemK-Shae coding sequences were commercially synthesized and cloned into pETDuet-1. Sequences of *cat194* cassette containing variable number of UAUU and UACAU (Sequences S1) were synthesized (GenScript) and cloned into pCN51-MazF-Sa using ApaI and XhoI restriction sites resulting in different variants of pCN51CAM-pemK-Sa1 and pCN51CAM-mazF-Sa. Plasmid constructs used in this study and PCR primers used for their preparation are summarized in Table 2.

**TABLE 2:** Plasmids and primers used in the study^*a*^

Plasmid construct(s)	Primers
pETDuet-pemK-Sa6	F: CCAGGATCCG**GTAAAACAATTTGATGTAATTAAAG** R: CTTGTCGAC**TTATATTGTTTCAATGTGTGC**
pCN51-pemK-Sa6	F: CCGGTCGAC*TAA*C*TAA*C*TAA*AGATTGGGAAGAAAGAGTTGGCCGAG AAAT**ATGGTAAAACAATTTGATGTA** R: CGCGGATCC**TTATATTGTTTCAATGTGTGC**
pCN51-mazF-Sa	F: CAGGTCGAC*TAA*C*TAA*C*TAA*TCTTCTAATTCAACGAATGA**ATGATTAG** R: CGGGGATCC**TTAATTTTTCTGGTGAGCTACTG**
pETDuet/pCN51, pemK-Sa6-E19R, pemK-Sa6-F42T, pemK-Sa6-E19RF42T	E19R-F: GCTAAAAGT**CGT**TTATATGTAGTTGTAAGTAATGATTATG E19R-R: CATATAA**ACG**ACTTTTAGCTTCATCTGGATTATTTAC F42T-F: TATCCAGTC**ACA**CAAAGAGATAGAATATATTTAACAG F42T-R: ATCTCTT**TGT**GTGACTGGATATGTCCAAATATAGGG
pETDuet-pemK-Sa6_6/pemK-Shae, cat194 in pCN51CAM-mazF-Sa/pemK-Sa1	GenScript
‍pALCP2G-pemIK-Sa1	Bukowski et al. (37)

^*a*^ Highlighted fragments: complementary to the coding sequence (bold); restriction sites (double underlined); extra stop codons (italic); mutated codons (bold and underlined); RBS-containing sequence preceding *pemK-Sa1* coding sequence (underlined). Restriction sites used for cloning: pETDuet, BamHI/SalI; pCN51, SalI/BamHI.

### Production and purification of PemK-Sa6 and its mutants

N-terminally 6xHis-tagged PemK-Sa1 and PemK-Sa6 and their variants were produced in *E. coli* BL21 (DE3) Gold and purified as described in a prior study (37). In brief, PemK-Sa6 or variants were purified in native conditions on HisPur Ni-NTA Superflow Agarose (Thermo Scientific). The protein expression was induced with 1 mM IPTG (A&A Biotechnology) at an OD_600_ of 0.6–0.8 and was carried out for 3 h at 30°C. PemK-Sa1 was expressed similar to PemK-Sa6, purified in denaturing conditions and renatured by dialysis into 50 mM NaH_2_PO_4_, pH 8.0, containing 300 mM NaCl and 20% glycerol. Both proteins were further purified by gel filtration on a Superdex 75 10/300 GL column (Cytiva) in 50 mM NaH_2_PO_4_, pH 8.0, containing 100 mM NaCl.

### Growth inhibition assays

Dilutions (1:100) of overnight cultures were grown to an OD_600_ of 0.6. Drops (10 µL) of serial dilutions (10^0^–10^4^) were placed on solid media containing relevant inducers: 50 µM IPTG or 2.5 µM CdCl_2_ for *E. coli* and *S. aureus*, respectively. The plates were incubated overnight. For suspension culture assays, overnight cultures were diluted to an OD_600_ of 0.02, and 200 µL aliquots were transferred to 96-well plates. The plates were incubated, and growth was monitored at 5-min intervals using a Sunrise microplate reader (Tecan) with temperature control and 5-s shaking before each measurement. After 2.5 h, inducers were added to final concentrations of 1 mM IPTG or 2.5 µM CdCl_2_, and growth was further monitored.

### Chloramphenicol minimal inhibitory concentration (MIC) assay

The pCN51, pCN51CAM-pemK-Sa1, pCN51CAM-mazF-Sa, pALCP2G, and pALCP2G-pemIK-Sa1 plasmids were transformed to *S. aureus* RN4220 strain (67). The assay was carried out in Mueller-Hinton Broth (MHB) in 96-well plates in a total volume of 100 µL. Each well contained 5.0 × 10^4 CFU of bacteria, CdCl_2_ at a final concentration of 125 nM (if needed), and chloramphenicol in concentrations ranging from 10 to 200 µg/mL in 10 µg/mL increments. Ninety-six-well plates were subsequently incubated in 37°C for 18 h. MIC values were assessed based on turbidity, where lack of turbidity was interpreted as growth inhibition. MIC values were determined in triplicate.

### Growth competition assay

Prior to the assay, *S. aureus* RN4220 was labeled with fluorescent reporters through the chromosomal incorporation of genes encoding a cyan fluorescent protein (CFP) or mCherry, as previously described (71). The bacteria were transformed with the plasmid pALCP2G-pemIK-Sa1, which encodes the PemIK-Sa1 TA system under its native promoter, or with a control plasmid (pALCP2G) (37). The transformants were grown overnight in tryptic soy broth (TSB) supplemented with chloramphenicol at 10 µg/mL. Then, the bacteria were collected by centrifugation and resuspended in TSB, and co-cultures were prepared by mixing the diluted bacteria (107 CFU/mL) in equal proportions. This resulted in the following pairs: *S. aureus*-CFP + pALCP2G and *S. aureus*-mCherry + pALCP2G-pemIK-Sa1, and *S. aureus*-CFP + pALCP2G-pemIK-Sa1 and *S. aureus*-mCherry + pALCP2G. The co-cultures were grown in media without antibiotics or supplemented with chloramphenicol at 80 µg/mL. Samples were taken at 0, 2.5, 5, 7.5, 24, and 48 h, then serially diluted and plated on tryptic soy agar (TSA) and TSA with chloramphenicol (10 µg/mL) using a spiral plater (Eddy Jet 2, IUL Instruments) in Log Mode 50 µL. After overnight incubation, the plates were scanned (for CFP: Cy2, 532/28 nm, and for mCherry: Cy3, 602/50 nm, ChemiDoc MP Imaging System, Bio-Rad), colonies were counted, and CFU per milliliter for each strain was calculated according to the spiral plater protocol. Finally, the fraction of bacteria carrying the PemIK-Sa1 system was determined for each co-culture, and the statistical significance of the results was assessed using the Mann-Whitney U test.

### RNAse activity assays

The RNase assays with phage MS2 RNA were performed as described elsewhere (37). In brief, 10 pmol of toxin protein was incubated for a specific period with 1 µg of MS2 RNA at 37°C in 10 mM Tris-HCl, pH 8.0, containing 10 mM EGTA in a total volume of 10 µL. Samples were separated in 1% agarose in Tris, acetate, and EDTA (TAE) buffer supplemented with SimplySafe dye (Eurx). Digestion of *cat194* transcripts was carried out as described elsewhere (37). The transcripts were obtained on the template of pCN51CAM-based constructs using TranscriptAid T7 High Yield Transcription Kit (Thermo Scientific). Short fluorescently labeled RNA substrates (GenScript) contained 6-carboxyfluorescein (6-FAM) at the 5′-end and 5-carboxytetramethylrhodamine (5-TAMRA) at the 3′-end. The assays were carried out in 50 mM Tris-HCl, pH 8.0, containing 150 mM NaCl and 70 nM of toxin proteins, in a total volume of 100 µL in 96-well black plates. After 1 hour, fluorescence was measured in a Synergy H1 microplate reader (BioTek) with excitation/emission at 495/519 nm. The following 6-FAM/5-TAMRA-labeled oligo RNAs were utilized: oligo-1, GUAUUG; oligo-2, AUAUUAC; and oligo-3, UACAUAU. Sequences recognized by PemK-Sa1 (UAUU) and MazF-Sa (UACAU) are underlined.

### Identification of type II toxin–antitoxin systems and drug resistance determinants in *S. aureus* genomes

Initially, 78,372 staphylococcal genome assemblies (NCBI taxonomy taxid 1279, including all subtaxa) obtained on 29 March 2023\ from NCBI GenBank were searched for operons encoding potential type II toxin–antitoxin pairs using methods described previously (34). Protein sequences representing 26 homology classes of TA pairs were retrieved from databases (Sequences S2 and S3). Protein sequences (4,750) coding for antibiotic resistance determinants (protein homolog model data set) were obtained on 29 March 2023 from CARD database (72). These sequences were used to query 75,919 *S*. *aureus* genome assemblies (NCBI Taxonomy species taxid 1280) using the translated BLAST tool (tblastn). The results were filtered based on query coverage (≥90%) and sequence similarity (≥80%). Only hits without internal stop codons were further analyzed. In the case of overlapping hits, only those with the highest sequence similarity were preserved. The search and preliminary results filtering was achieved using a Nextflow (73) pipeline run in a Miniconda environment that included the following packages: nextflow 22.10.6, blast 2.14.1 (74), python 3.11.3, numpy 1.26.3 (75), and pandas 2.2.0 (76).

### Analysis of co-occurrence of type II toxin–antitoxin systems with drug resistance determinants

A TA system operon was labeled as identified when the antitoxin coding sequence was located in one contig and on the same strand as well as within 100 bp distance with respect to the toxin coding sequence. Co-occurrence of a TA system with a drug resistance determinant was identified when both were found among contigs of a single genome assembly. The probability of random co-occurrence was calculated for every possible TA system/resistance determinant pair using hypergeometric distribution. To analyze biases in co-occurrence, each genetic element of a given pair needed a high enough prevalence to have a chance of co-occurring with another genetic element on a purely random basis. Therefore, the pairs for which the probability of co-occurring at least 10 times was lower than 99% were not further analyzed. For the remaining pairs, two ratios were calculated: the number of genomes carrying a drug resistance determinant and a TA system (R+TA) to the number of genomes carrying the given TA system (TA), denoted as (R+TA)/TA; the number of genomes carrying the given drug resistance determinant (R) to the number of all genomes (ALL), denoted as R/ALL. Next, the ratios of these ratios were calculated (the higher value ratio to the lower value ratio) to determine how many times more or less often a drug resistance determinant occurs among genomes carrying a given TA system compared to its general occurrence among all genomes. The described analysis of co-occurrence and results visualization were carried out in Jupyter Lab (77) in a Miniconda environment that included the following packages: jupyterlab 4.0.12, scipy 1.10.1 (78), python 3.11.3, numpy 1.26.3, pandas 2.2.0, and matplotlib 3.7.1 (79).

### Phylogenetic relations among PemK-Sa6 and other PemK homologs in staphylococci

Phylogenetic relationships among PemK-Sa6 and other staphylococcal PemK homologs were determined by aligning six unique sequences of PemK-Sa6 toxins encoded in staphylococcal genomes and representative sequences of 15 remaining PemK homologs. Columns containing gaps were removed from the alignment, which was then used to create a phylogenetic tree based on the Whelan and Goldman (WAG) model. The alignment and the tree were generated in CLC Main Workbench 23 (Qiagen).

## Data Availability

Staphylococcal genomes were obtained from the publicly available NCBI GenBank database, and their accession version numbers are listed in Table S1. Protein sequences of drug resistance determinants were obtained from the publicly available CARD database (protein homolog model data set). Other input sequences are provided in the supplemental files antitoxins.faa and toxins.faa. Tables S1 to S6 contain raw numeric output data. Code for data analysis and visualization is provided in GitHub repositories tblastn-genomes, mlst-genomes, and tas-drug at https://github.com/michalbukowski.

## SUPPLEMENTAL MATERIAL

Sequences S1 msystems.00957-24-s0001.pdf Cat194 nucleotide sequences encoding a chloramphenicol resistance determinant.

Sequences S2 msystems.00957-24-s0002.pdf Amino acid sequences of antitoxins.

Sequences S3 msystems.00957-24-s0003.pdf Amino acid sequences of toxins.

Supplemental figures msystems.00957-24-s0004.pdf Figure S1 to S10.

Supplemental material msystems.00957-24-s0005.pdf Information for Table S1 and drug resistance names.

Supplemental tables msystems.00957-24-s0006.xlsx Tables S2 to S7.

ASM does not own the copyrights to Supplemental Material that may be linked to, or accessed through, an article. The authors have granted ASM a non-exclusive, world-wide license to publish the Supplemental Material files. Please contact the corresponding author directly for reuse.
