# Supplementary material for: Analysis of co-occurrence of type II toxin–antitoxin systems and antibiotic resistance determinants in Staphylococcus aureus
Source: mSystems. 2025 Feb 27;10(3):e00957-24. doi: 10.1128/msystems.00957-24 (PMC11915791; doi:10.1128/msystems.00957-24)
Supplement: Sequences S1 — Cat194 nucleotide sequences encoding a chloramphenicol resistance determinant. [file msystems.00957-24-s0001.pdf]

Supplemental sequences S1. Cat194 nucleotide sequences encoding a chloramphenicol resistance determinant. The variants contain a certain number of UACAU and UAUU sequences in their transcripts, which are recognized by MazF-Sa and PemK-Sa1 toxins, respectively.

>**cat194 wild type cassette**, number of UACAU and UAUU in the transcript: 1, 13 (respectively)
GGGCCCACCTAGGTATTATCAAGATAAGAAAGAAAAGGATTTTTCGCTACGCTCAAATCC
TTTAAAAAAACACAAAAGACCACATTTTTTAATGTGGTCTTTTATTCTTCAACTAAAGCA
CCCATTAGTTCAACAAACGAAAATTGGATAAAGTGGGATATTTTTAAAATATATATTTAT
GTTACAGTAATATTGACTTTTAAAAAAGGATTGATTCTAATGAAGAAAGCAGACAAGTAA
GCCTCCTAAATTCACTTTAGATAAAAATTTAGGAGGCATATCAAATGAACTTTAATAAAA
TTGATTTAGACAATTGGAAGAGAAAAGAGATATTTAATCATTATTTGAACCAACAAACGA
CTTTTAGTATAACCACAGAAATTGATATTAGTGTTTTATACCGAAACATAAAACAAGAAG
GATATAAATTTTACCCTGCATTTATTTTCTTAGTGACAAGGGTGATAAACTCAAATACAG
CTTTTAGAACTGGTTACAATAGCGACGGAGAGTTAGGTTATTGGGATAAGTTAGAGCCAC
TTTATACAATTTTTGATGGTGTATCTAAAACATTCTCTGGTATTTGGACTCCTGTAAAGA
ATGACTTCAAAGAGTTTTATGATTTATACCTTTCTGATGTAGAGAAATATAATGGTTCGG
GGAAATTGTTTCCCAAAACACCTATACCTGAAAATGCTTTTTCTCTTTCTATTATTCCAT
GGACTTCATTTACTGGGTTTAACTTAAATATCAATAATAATAGTAATTACCTTCTACCCA
TTATTACAGCAGGAAAATTCATTAATAAAGGTAATTCAATATATTTACCGCTATCTTTAC
AGGTACATCATTCTGTTTGTGATGGTTATCATGCAGGATTGTTTATGAACTCTATTCAGG
AATTGTCAGATAGGCCTAATGACTGGCTTTTATAATATGAGATAATGCCGACTGTACTTT
TTACAGTCGGTTTTCTAATGTCACTAACCTGCCCCGTTAGTTGAAGAAGGCCGCGGCCTC
GAG

>**cat194_mazF_min**, number of UACAU in the transcript: 0
ATTTAAATGGGCCCCATATGACCTAGGTATTATCAAGATAAGAAAGAAAAGGATTTTTCG
CTACGCTCAAATCCTTTAAAAAAACACAAAAGACCACATTTTTTAATGTGGTCTTTTATT
CTTCAACTAAAGCACCCATTAGTTCAACAAACGAAAATTGGATAAAGTGGGATATTTTTA
AAATATATATTTATGTTACAGTAATATTGACTTTTAAAAAAGGATTGATTCTAATGAAGA
AAGCAGACAAGTAAGCCTCCTAAATTCACTTTAGATAAAAATTTAGGAGGCATATCAAAT
GAACTTTAATAAAATTGATTTAGACAATTGGAAGAGAAAAGAGATATTTAATCATTATTT
GAACCAACAAACGACTTTTAGTATAACCACAGAAATTGATATTAGTGTTTTATACCGAAA
CATAAAACAAGAAGGATATAAATTTTACCCTGCATTTATTTTCTTAGTGACAAGGGTGAT
AAACTCAAATACAGCTTTTAGAACTGGTTACAATAGCGACGGAGAGTTAGGTTATTGGGA
TAAGTTAGAGCCACTTTATACAATTTTTGATGGTGTATCTAAAACATTCTCTGGTATTTG
GACTCCTGTAAAGAATGACTTCAAAGAGTTTTATGATTTATACCTTTCTGATGTAGAGAA
ATATAATGGTTCGGGGAAATTGTTTCCCAAAACACCTATACCTGAAAATGCTTTTTCTCT
TTCTATTATTCCATGGACTTCATTTACTGGGTTTAACTTAAATATCAATAATAATAGTAA
TTACCTTCTACCCATTATTACAGCAGGAAAATTCATTAATAAAGGTAATTCAATATATTT
ACCGCTATCTTTACAGGTACACCATTCTGTTTGTGATGGTTATCATGCAGGATTGTTTAT
GAACTCTATTCAGGAATTGTCAGATAGGCCTAATGACTGGCTTTTATAATATGAGATAAT
GCCGACTGTACTTTTTACAGTCGGTTTTCTAATGTCACTAACCTGCCCCGTTAGTTGAAG
AAGGCCGCGGCCTCGAGATTTAAAT

>**cat194_mazF_max**, number of UACAU in the transcript: 2
ATTTAAATGGGCCCCATATGACCTAGGTATTATCAAGATAAGAAAGAAAAGGATTTTTCG
CTACGCTCAAATCCTTTAAAAAAACACAAAAGACCACATTTTTTAATGTGGTCTTTTATT
CTTCAACTAAAGCACCCATTAGTTCAACAAACGAAAATTGGATAAAGTGGGATATTTTTA
AAATATATATTTATGTTACAGTAATATTGACTTTTAAAAAAGGATTGATTCTAATGAAGA
AAGCAGACAAGTAAGCCTCCTAAATTCACTTTAGATAAAAATTTAGGAGGCATATCAAAT
GAACTTTAATAAAATTGATTTAGACAATTGGAAGAGAAAAGAGATATTTAATCATTATTT
GAACCAACAAACTACATTTAGTATAACCACAGAAATTGATATTAGTGTTTTATACCGAAA
CATAAAACAAGAAGGATATAAATTTTACCCTGCATTTATTTTCTTAGTGACAAGGGTGAT
AAACTCAAATACAGCTTTTAGAACTGGTTACAATAGCGACGGAGAGTTAGGTTATTGGGA
TAAGTTAGAGCCACTTTATACAATTTTTGATGGTGTATCTAAAACATTCTCTGGTATTTG
GACTCCTGTAAAGAATGACTTCAAAGAGTTTTATGATTTATACCTTTCTGATGTAGAGAA

ATATAATGGTTCGGGGAAATTGTTTCCCAAAACACCTATACCTGAAAATGCTTTTTCTCT
TTCTATTATTCCATGGACTTCATTTACTGGGTTTAACTTAAATATCAATAATAATAGTAA
TTACCTTCTACCCATTATTACAGCAGGAAAATTCATTAATAAAGGTAATTCAATATATTT
ACCGCTATCTTTACAGGTACATCATTCTGTTTGTGATGGTTATCATGCAGGATTGTTTAT
GAACTCTATTCAGGAATTGTCAGATAGGCCTAATGACTGGCTTTTATAATATGAGATAAT
GCCGACTGTACTTTTTACAGTCGGTTTTCTAATGTCACTAACCTGCCCCGTTAGTTGAAG
AAGGCCGCGGCCTCGAGATTTAAAT

>**cat194_pemK_min**, number of UAUU in the transcript: 0
ATTTAAATGGGCCCCATATGACCTAGGTATTATCAAGATAAGAAAGAAAAGGATTTTTCG
CTACGCTCAAATCCTTTAAAAAAACACAAAAGACCACATTTTTTAATGTGGTCTTTTATT
CTTCAACTAAAGCACCCATTAGTTCAACAAACGAAAATTGGATAAAGTGGGATATTTTTA
AAATATATATTTATGTTACAGTAATATTGACTTTTAAAAAAGGATTGATTCTAATGAAGA
AAGCAGACAAGTAAGCCTCCTAAATTCACTTTAGATAAAAATTTAGGAGGCATATCAAAT
GAACTTTAATAAAATTGATTTAGACAATTGGAAGAGAAAAGAGATCTTTAATCATTACTT
GAACCAACAAACGACTTTTAGTATAACCACAGAAATTGATATAAGTGTTTTATACCGAAA
CATAAAACAAGAAGGATATAAATTTTACCCTGCATTTATCTTCTTAGTGACAAGGGTGAT
AAACTCAAATACAGCTTTTAGAACTGGTTACAATAGCGACGGAGAGTTAGGTTACTGGGA
TAAGTTAGAGCCACTTTATACAATTTTTGATGGTGTATCTAAAACATTCTCTGGTATATG
GACTCCTGTAAAGAATGACTTCAAAGAGTTTTATGATTTATACCTTTCTGATGTAGAGAA
ATATAATGGTTCGGGGAAATTGTTTCCCAAAACACCTATACCTGAAAATGCTTTTTCTCT
TTCTATCATTCCATGGACTTCATTTACTGGGTTTAACTTAAATATCAATAATAATAGTAA
TTACCTTCTACCCATCATTACAGCAGGAAAATTCATTAATAAAGGTAATTCAATATACTT
ACCGCTATCTTTACAGGTACATCATTCTGTTTGTGATGGTTATCATGCAGGATTGTTTAT
GAACTCTATCCAGGAATTGTCAGATAGGCCTAATGACTGGCTTTTATAATATGAGATAAT
GCCGACTGTACTTTTTACAGTCGGTTTTCTAATGTCACTAACCTGCCCCGTTAGTTGAAG
AAGGCCGCGGCCTCGAGATTTAAAT

>**cat194_pemK_max**, number of UAUU in the transcript: 26
ATTTAAATGGGCCCCATATGACCTAGGTATTATCAAGATAAGAAAGAAAAGGATTTTTCG
CTACGCTCAAATCCTTTAAAAAAACACAAAAGACCACATTTTTTAATGTGGTCTTTTATT
CTTCAACTAAAGCACCCATTAGTTCAACAAACGAAAATTGGATAAAGTGGGATATTTTTA
AAATATATATTTATGTTACAGTAATATTGACTTTTAAAAAAGGATTGATTCTAATGAAGA
AAGCAGACAAGTAAGCCTCCTAAATTCACTTTAGATAAAAATTTAGGAGGCATATCAAAT
GAACTTTAATAAAATTGATTTAGACAATTGGAAGAGAAAAGAGATATTTAATCATTATTT
GAACCAACAAACGACTTTTAGTATTACCACAGAAATTGATATTAGTGTATTATACCGAAA
TATTAAACAAGAAGGATATAAATTTTACCCTGCATTTATTTTCTTAGTGACAAGGGTTAT
TAACTCAAATACAGCTTTTAGAACTGGTTACAATAGCGACGGAGAGTTAGGTTATTGGGA
TAAGTTAGAGCCACTTTATACTATTTTTGATGGTGTATCTAAAACATTCTCTGGTATTTG
GACTCCTGTAAAGAATGACTTCAAAGAGTTTTATGATTTATATTTATCTGATGTAGAGAA
ATATAATGGTTCGGGGAAACTATTTCCCAAAACACCTATTCCTGAAAATGCTTTTTCTCT
TTCTATTATTCCATGGACTTCATTTACTGGGTTTAACTTAAATATTAATAATAATAGTAA
TTATTTGCTACCTATTATTACAGCAGGAAAATTTATTAATAAAGGTAATTCTATTTATTT
ACCGCTATCTTTACAGGTACATCATTCTGTTTGTGATGGTTATCATGCAGGACTATTTAT
GAACTCTATTCAGGAATTGTCAGATAGGCCTAATGACTGGCTATTATAATATGAGATAAT
GCCGACTGTACTTTTTACAGTCGGTTTTCTAATGTCACTAACCTGCCCCGTTAGTTGAAG
AAGGCCGCGGCCTCGAGATTTAAAT
