# Supplementary material for: Analysis of co-occurrence of type II toxin–antitoxin systems and antibiotic resistance determinants in Staphylococcus aureus
Source: mSystems. 2025 Feb 27;10(3):e00957-24. doi: 10.1128/msystems.00957-24 (PMC11915791; doi:10.1128/msystems.00957-24)
Supplement: Sequences S2 — Amino acid sequences of antitoxins. [file msystems.00957-24-s0002.pdf]

Supplementary sequences S2. Amino acid sequences of antitoxins used as a query to identify type II toxin-antitoxin systems in Staphylococcus aureus genomes using the translated BLAST tool (tblastn).

>MazE-Sa
MLSFSQNRSHSLEQSLKEGYSQMADLNLSLANEAFPIECEACDCNETYLSSNSTNE
>PemI-Sa1
MAKSLKDLNKTSERVFKNGNSKAISLSKKTIRLANFDIGDTVEVQKMNGGLFITKKKESI
EDRIKNFFQSGGKYTELEVDWEERVGREI
>PemI-Sa2
MSNTAKIRRQGGATVFSIPPALLKMLGVEVGAELTLVVNNGSLVATPKQGKKRYTMAEML
EGANELVALNKEAASWNVSAPVGNEVF
>PemI-Sa3
MHTTRLKRVGGSVMLTVPPALLNALSLGTDNEVGMVIDNGRLIVEPYRRPQYSLAELLAQ
CDPNAEISAEEREWLDAPATGQEEI
>PemI-Sa4
MLTTKSRKQGSSVVLTLPSNNGQKPKADQEYIVMYSDDGTITLVPKLQDPFSGGSEGEYY
EKDEWYDIEPGGRELF
>PemI-Sa5
MVMLSIKKWGNSNGLRLPKSVMEYLQIHTEDKVKITQEESNGKKRLIIEAVDSDNDLTIE
QLFENYKEEKVHVTIQDLGNAVGNEKW
>PemI-Sa6
MSKINSKIFKYGNSQAISLSKEALHEAGLEIGDVLEYYIEEDNKILFKKVEERSYKDFIK
EYYKHGGRYMDEKINQEADEGK
>PemI-Sagn
MVKTQSRIIRNGNSQALTLSKYMMEKSGLKLGDHLDYHVKNGKIIFEKSKEDQFEQKFKE
FFENGGTYEDYRVDEVESAGREQW
>PemI-Sgal
MFQYKSKVMKYGNGQAISISKEIMEKAGLNIGDKLEIESADQHRIVFNKVEEESLKEKIQ
TFYKNGGKNKT
>PemI-Svit
MSEPTNKAYAKVYKSGNGQVISIKKDMLEKAGFKIGDELVMNVKGNQIVLEKPNTFKDRW
RKFIEDGGEYERGEYDWGESVGREEW
>PemI-Ssap
MDCVKKSQAKVYKSGNGQVISVRKRDLERAGFQIGDNLDVEVSNSQISLIKSHTFQSEWQ
SFIENGGQYERSEYDWRN
>PemI-Shae
MNSAKVFKNGNSQAISLNKQILKQADLEIGDDLTVYVTNDGKVVFSKKELSIKEQIHNYY
KNGGIYSEDEIDYGQDVGKEKW
>PemI-Scar
MSENRTHIFKTGQEHAVILPEPIIKSLNLKPGDVLVQEIVDGQIILKKEKHQSFSEEWNQ
FFEQGGTYDDYETHQWGEASEREKW
>PemI-Swar
MKTTYAKIYKSGNGQAISLKKNILQQVGLKVGDDIEVKVKSGQIVLTKPNSFKEKWRDFV
ESGGKYDHNKYDWGQSVGRELW
>PemI-Scap
MSTLSKVIRSGNSQAITLNKTILNEAGLNIGDNLSVEVKDGEIKFTKKNKSIIDEIHDFY
RNGGYYDEEEIDFGKSVGRESW
>PemI-Ssci
MDKVKTRKQGNAVMVTIAKKFNVPEGQEFYITKEADGTIALIPKIQDYFKNVTVGEFIDE
EDSLAQNFRTVGNELDE
>PemI-Smic
MVTTQSKIFKNGNSQAISLNKTLMQQAGFHIGDELDVTIQEEALLFTKKNKTIKDEIQDF
YKNGGKYTESEIDYGESRGSELW
>YefM-Sa1
MIIKNYSYARQNLKALMTKVNDDSDMVTVTSTDDKNVVIMSESDYNSMMETLYLQQNPNN
AEHLAQSIADLERGKTITKDIDV
>YefM-Sa2

```
MIITSPTEARKDFYQLLKNVNNNHEPIYISGNNAENNAVIIGLEDWKSIQETIYLESTGT
MDKVREREKDNSGTTNIDDIDWDNL
>YefM-Sepi
MVMEAVLYSTFRNHLKDYMKKVNDEFEPLTVVNKNPDEDIVVLSKSEWDSIQETLRIAQN
KELSDKVLRGMAQVRAGGTQVHVIEE
>DinJ
MATTKKKPIHVNVDENLKEEAEQLFDDLGLNMTSAITIFLKQSINEQAIPFMINKGNKET
LQALKDIKEGNVHGGFSSVEDLMENLNA
>Omega
MIVGNLGAQKAKRNDTPISAKKDIMGDKTVRVRADLHHIIKIETAKNGGNVKEVMDQALE
EYIRKYLPDKL
>Epsilon
MAVTYEKTFEIEIINELSASVYNRVLNYVLNHELNKNDSQLLEVNLLNQLKLAKRVNLFD
YSLEELQAVHEYWRSMNRYSKQVLNKEKVA
>PezTAnti
MIGQNIKSLRRTNHLTQPEFAKMVNISRNSLSRYENGTSSVSTDLIDRICQKFNVSYVDI
VGEEKMLTPVEDYQLTLKIEVIKERGSSILSQLYRYQDSQGIAFDDDANPWIIMSDDISD
LINMKIYLVDTFEEIERYNGYLDGIERMLKHAMKQAVVS
>MazE-Sa2
MFLINQRTTRDNSHNVGLEIISHNVKLKPIIYQPKVSEYGSLSFNNKIIGGKTVEDLPIA
LGSVREKTKTEQERKEELKVLFNRTRDRYDKTFRDLVNL
>MazE-Sepi2
MEGLQMITTRKLRKAGNSSVVSVPTEVIAALGISNGDNLKFNVKDNKVTIEKEVREDEEF
FKLLDETFTEYNQALKRMVDL
>MazE-Slen
MEILNVKKIRKVGNSSVITIPKNVMEAINIHEGDAVEFIEEDNKIWLKPSKSINEHDDIL
KLADEISNKYDDVFKGLVDR
```
