# Supplementary material for: Analysis of co-occurrence of type II toxin–antitoxin systems and antibiotic resistance determinants in Staphylococcus aureus
Source: mSystems. 2025 Feb 27;10(3):e00957-24. doi: 10.1128/msystems.00957-24 (PMC11915791; doi:10.1128/msystems.00957-24)
Supplement: Sequences S3 — Amino acid sequences of toxins. [file msystems.00957-24-s0003.pdf]

**Supplementary sequences S3.** Amino acid sequences of toxins used as a query to identify type II toxin-antitoxin systems in Staphylococcus aureus genomes using the translated BLAST tool (tblastn).

>MazF-Sa
MIRRGDVYLADLSPVQGSEQGGVRPVVIIQNDTGNKYSPTVIVAAITGRINKAKIPTHVE
IEKKKYKLDKDSVILLEQIRTLDKKRLKEKLTYLSDDKMKEVDNALMISLGLNAVAHQKN
>PemK-Sa1
MNIKQFDILYIDLNPTRGREKHNVRPCLVINNQMSIDGTNFVWVLPITTRGLRYPTDIQL
KTKKGLVSGVIDTVQIRALDLKARQYNYKDELQDNLKNDILKAIKTYLKPTL
>PemK-Sa2
MVRSNIPKRGDVFLVDLNPVVGNEMRDAHRCVVITPHEINMVGMCLTVPITTGGAFTRKV
GLAVNISGHKTTGVALCNQVRALDILERMKQKTAKHIDTLDASTTDEIVARVVSMIDPA
>PemK-Sa3
MERGEIWLVSLDPTAGHEQQGTRPVLIVTPAAFNRVTRLPVVVPVTSGGNFARTAGFAVS
LDGVGIRTTGVVRCDQPRTIDMKARGGKRLERVPETIMNEVLGRLSTILT
>PemK-Sa4
MSDEKKYIPKKGDIVWIDFDPSAGKEIQKRRPGLVVSRYEFNRKTMFAVICPITSTIKNM
PTRYTLPDEMETHGQVVISQLKSLDFTERKLSQIEHLPLKDMAKIDQIIEYIF
>PemK-Sa5
MVKVPHQGDILLLNTAPRSGHEQTGKRPYIVLSHDIIADYSNVVIVAPISSTKRNYPLYV
SINPSYGMKTSGKVLLDQLTTIDYEARQCVFLETAHEKLIDELLLKVRTVFQKVNKTNKF
>PemK-Sa6
MVKQFDVIKVNNPDEAKSELYVVVSNDYVNSNSPYIWTYPVFQRDRIYLTDIELVTKRYN
YYGVIDCGIIGHIDLTKKEIKVLDKLQPRIQNKLMESIQAHIETI
>PemK-Sagn
MVNQYSIIRVNLNPTKGREKGKYRPCIVLSATYYNLATGFVWAVPITSRDERYPTDIKLK
TKYNNITGIVDLAQIKTLDLKARDYELVDEASSLTVNAIKPILISLLCLK
>PemK-Sgal
MVKQFDIISVNNDDYFIVVSNNLVNQNSDTVWVMPINNNEPKYVTDILLATKKKLIKGII
DTTQISHLIIEQSNYKVLDHAQPRICKDIVDAIEAHMEIL
>PemK-Svit
MVKQYDIIKLNLNPVQGNEKGNYRPCLVVNITEFTKQSGFAWVIPITSRKQSRYPTDVIV
ETEKGLINGVVDCAHIRTVDLYTRSHQKVDVLKANKIEEVKDILTGIMDL
>PemK-Ssap
MVKQYDIIKLNFNTDKTSEVGDYRPCLVVSDNVFTKGSGFTWVMPILSGKEASYPTDVVV
ESKEDIVRGVIDSVHIHSFDLQTRDYHTIDVLSEHKVKEVKDILSGVLNM
>PemK-Shae
MVSQFDIIKIDLNPVKGIEKGKYRPCLIISNNYINQYTGIIWVMPITSRNKRYPSDIEVK
TKLGNISGIIDTVQIQSLDIQYRNYRVVDHLHENLKHQILETIEAHTEPI
>PemK-Scar
MVEQYKIIDVNLDPILGREKGKYRPCVVLSRTSFNNKTSLVWVSPITSRPVKYPTDVALK
TMEHHIKGTIDVGQIRTLDLSTRHYREVDSVSHEVMHKIDDIITNILKIESF
>PemK-Swar
MVNLYDVIKLDFNPVIGTEKGNYRPCLVISDTEFNKVSGFAWVIPITKRYDERYPTDVLV
KTKNQHINGFIDCTQIKSVDIHARPYRYLDISTTEKGIEVNDRLKSLLNL
>PemK-Scap
MVNQFDVVMIDLDPTRGKEKQKYRPCVVVSNDFVNQHSPFIWALPITNRPKRFPSDVTVK
TKNNSITGIIDTIQIRSLDAKARRIKKIDELDESIKSDVINTIVAHSCMI
>PemK-Ssci
MNSISQGTLIYIDFEPSKGSEIKKRRPAIVISRDEYNLASNLVIVCPITSTDKDRPYFVP
INNKNLKSNSKVNTKQVYSLDCTERAGRNIQIIGRITNKELMNIAQHFLMNFNFNF
>PemK-Smic
MVKQFDIITVDLNPSRGREKNKYRPCLIISNDLMNKNTPLSWIFPITNRPKKFPSDIILK
TNNKNVEGIIDTVQIRALDLTVRNAKVIDTLHDSLKATVLKTIEAHTEMV
>YoeB-Sa1
MARLNITFSPQAFEDYKYFQQNDKKMVKKINELLKSIDRNGALEGIGKPEKLKSNLTGYY
SRRINHEHRLVYTVDDNHIKIASCKYHY

```
>YoeB-Sa2
MSNYTVKIKNSAKSDLKKIKHSYLKKSFLEIVETLKNDPYKITQSFEKLEPKYLERYSRR
INHQHRVVYTVDDRNKEVLILSAWSHYD
>YoeB-Sepi
MLLKFTEDAWADYCYWQNQDKKTLKRINKLIKDIQRDPFVGMGKPEPLKYDYQGAWSRRI
DAENRLIYMMDGDSVAFLSFKDHY
>YafQ
MLEIFYTNQFKKDFKKAKKQGKNLEKLKEVIVLLQEQQTLPLKYKDHALTGNYIGTRECH
IEPDWLLIYKIDGDKLILTLARIGSHSELFR
>Zeta
MANIVNFTDKQFENRLNDNLEELIQGKKAVESPTAFLLGGQPGSGKTSLRSAIFEETQGN
VIVIDNDTFKQQHPNFDELVKLYEKDVVKHVTPYSNRMTEAIISRLSDQGYNLVIEGTGR
TTDVPIQTATMLQAKGYETKMYVMAVPKINSYLGTIERYETMYADDPMTARATPKQAHDI
VVKNLPTNLETLHKTGLFSDIRLYNREGVKLYSSLETPSISPKETLEKELNRKVSGKEIQ
PTLERIEQKMVLNKHQETPEFKAIQQKLESLQPPTPPIPKTPKLPGI
>PezTTox
MKLEAFSETQLQLALNRIIRSLTRGKTPSKHPRAILLGGQSGAGKTTLHRIKQKEFQGNI
VIIDGDSYRYLHPNYLAIQREYGKESVNYTKWFAGKMVEHLVCVLGKQGYHLLIEGTLRT
TEVPRKTARLLKSYGYKVSLALIATKPELSYLSTLIRYEELYAVDPNKARATSKAYHDNI
VRHLVGNLQELEDEKQFDQIQIYQRDKQCVYDSYKDKNGAAAVLQACLFGKWHTIEKEMM
RLGKEQLHLLRAQRDEQH
>FicDoc-Sa
MTEYLNYDDIVDINSWIIDIYSPNEPKIVISYEALDMAVQSPQQEVFGKVLYPTIEEKGA
NLYMNLATRHPFGNANKRTAFMSLDVFLQKNGKYIDVDTDEATLFTMRVVNENLDQATIV
RWIKRYIKDI
>FicDoc-Sepi2
MQSTKYLTEKQVIAINVKAIQDFSPKEQVGVKVPEVLNATIEGVKQSFGGVELYETIERK
AAFIYRNIAQKHAFHNANKRTAFTSMVIFLKLNSINFECTQDEAVQFTLRVVNDKNLTLE
GIETWIKRHCK
>FicDoc-Slen
MIRYLTEKEIITFNIYVIQKYSPKEPIGVVDATALNMLVNAPKQYVFGIEQYPTLALKAS
NLFRNLVKKHVFYNGNKRTAFICLNIFLNLNGYELEVNTKEAIDFTVMIATHDLQEDDIE
QWILKHIKIK
```
