# Supplementary material for: Analysis of co-occurrence of type II toxin–antitoxin systems and antibiotic resistance determinants in Staphylococcus aureus
Source: mSystems. 2025 Feb 27;10(3):e00957-24. doi: 10.1128/msystems.00957-24 (PMC11915791; doi:10.1128/msystems.00957-24)
Supplement: Supplemental figures — Figure S1 to S10. [file msystems.00957-24-s0004.pdf]

Analysis of co-occurrence of type II toxin-antitoxin systems and antibiotic resistance determinants in *Staphylococcus aureus*

Michał Bukowski[1], Michał Banasik[1#], Kinga Chlebicka[1], Katarzyna Bednarczyk[1], Emilia Bonar[1], Dominika Sokołowska[1], Tomasz Żądło[1,2], Grzegorz Dubin[3], Benedykt Władyka[1]*

[1]Jagiellonian University in Krakow, Faculty of Biochemistry, Biophysics and Biotechnology, Department of Analytical Biochemistry, Krakow, Poland; [2]Jagiellonian University in Krakow, Doctoral School of Exact and Natural Sciences, Krakow, Poland; [3]Jagiellonian University in Krakow, Malopolska Centre of Biotechnology, Krakow, Poland

[#]current affiliation: National Institute of Chemistry, Department of Synthetic Biology and Immunology, Hajdrihova 19, p.p. 660 SI-1001 Lublana, Slovenia

*corresponding author: benedykt.wladyka@uj.edu.pl

Supplementary figures

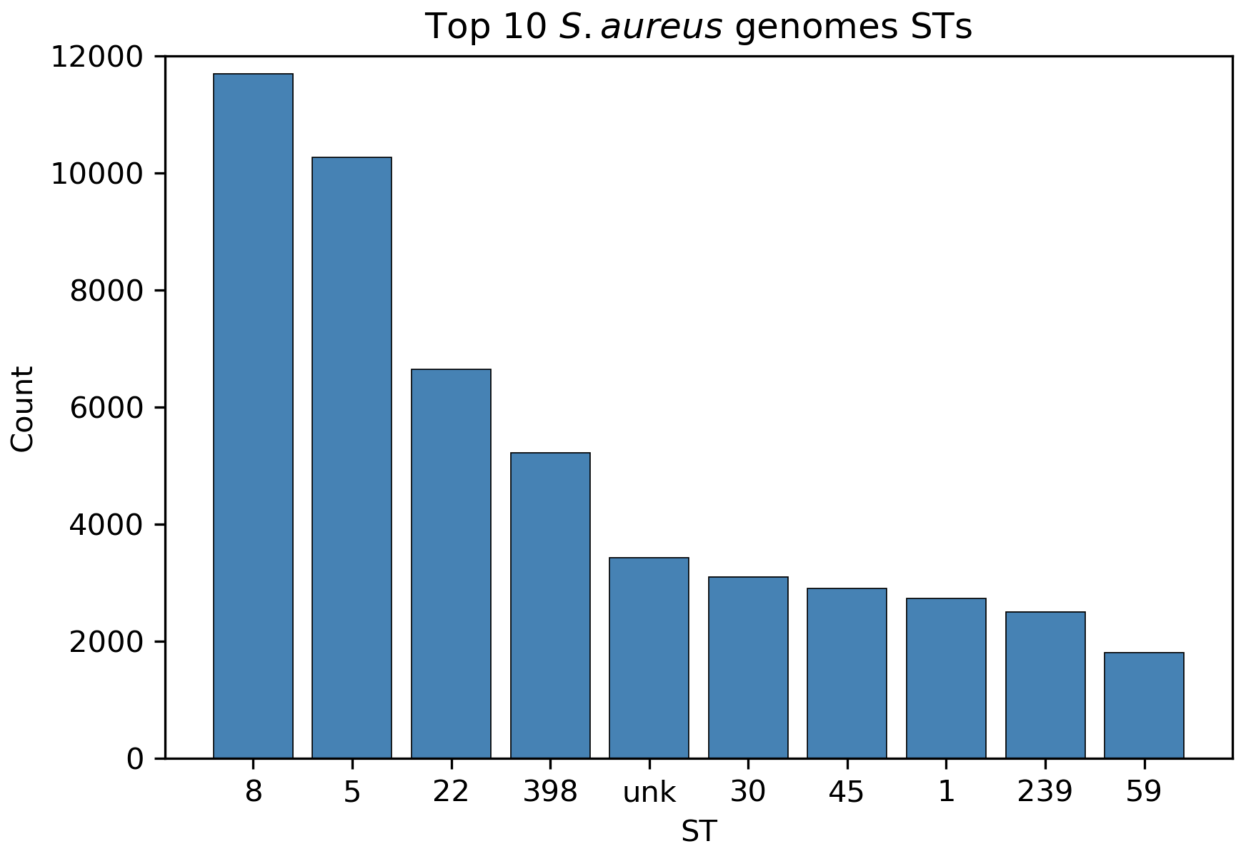

Fig. S1. Distribution of *Staphylococcus aureus* genomes among the 10 most numerous sequence types (STs). Four STs (8, 5, 22, and 398) encompass nearly 45% of *S. aureus* genomes deposited in NCBI GenBank. The top 9 STs encompass nearly 65% of genomes. There is less than 5% of genomes of indeterminable STs (unk).

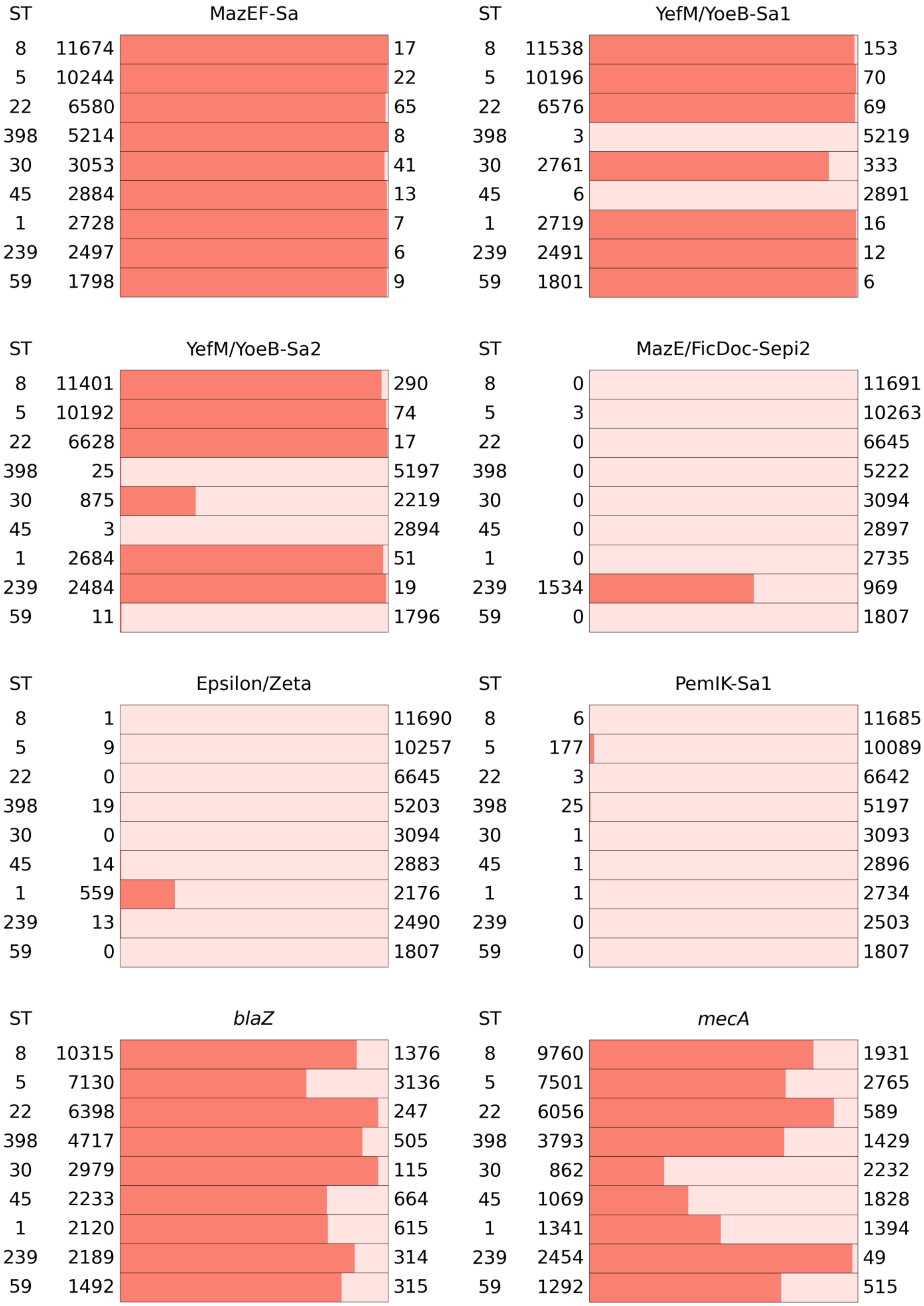

ST
MazEF-Sa
8 11674 17
5 10244 22
22 6580 65
398 5214 8
30 3053 41
45 2884 13
1 2728 7
239 2497 6
59 1798 9
ST
YefM/YoeB-Sa1
8 11538 153
5 10196 70
22 6576 69
398 3 5219
30 2761 333
45 6 2891
1 2719 16
239 2491 12
59 1801 6
ST
YefM/YoeB-Sa2
8 11401 290
5 10192 74
22 6628 17
398 25 5197
30 875 2219
45 3 2894
1 2684 51
239 2484 19
59 11 1796
ST
MazE/FicDoc-Sepi2
8 0 11691
5 3 10263
22 0 6645
398 0 5222
30 0 3094
45 0 2897
1 0 2735
239 1534 969
59 0 1807
ST
Epsilon/Zeta
8 1 11690
5 9 10257
22 0 6645
398 19 5203
30 0 3094
45 14 2883
1 559 2176
239 13 2490
59 0 1807
ST
PemIK-Sa1
8 6 11685
5 177 10089
22 3 6642
398 25 5197
30 1 3093
45 1 2896
1 1 2734
239 0 2503
59 0 1807
ST
blaZ
8 10315 1376
5 7130 3136
22 6398 247
398 4717 505
30 2979 115
45 2233 664
1 2120 615
239 2189 314
59 1492 315
ST
mecA
8 9760 1931
5 7501 2765
22 6056 589
398 3793 1429
30 862 2232
45 1069 1828
1 1341 1394
239 2454 49
59 1292 515

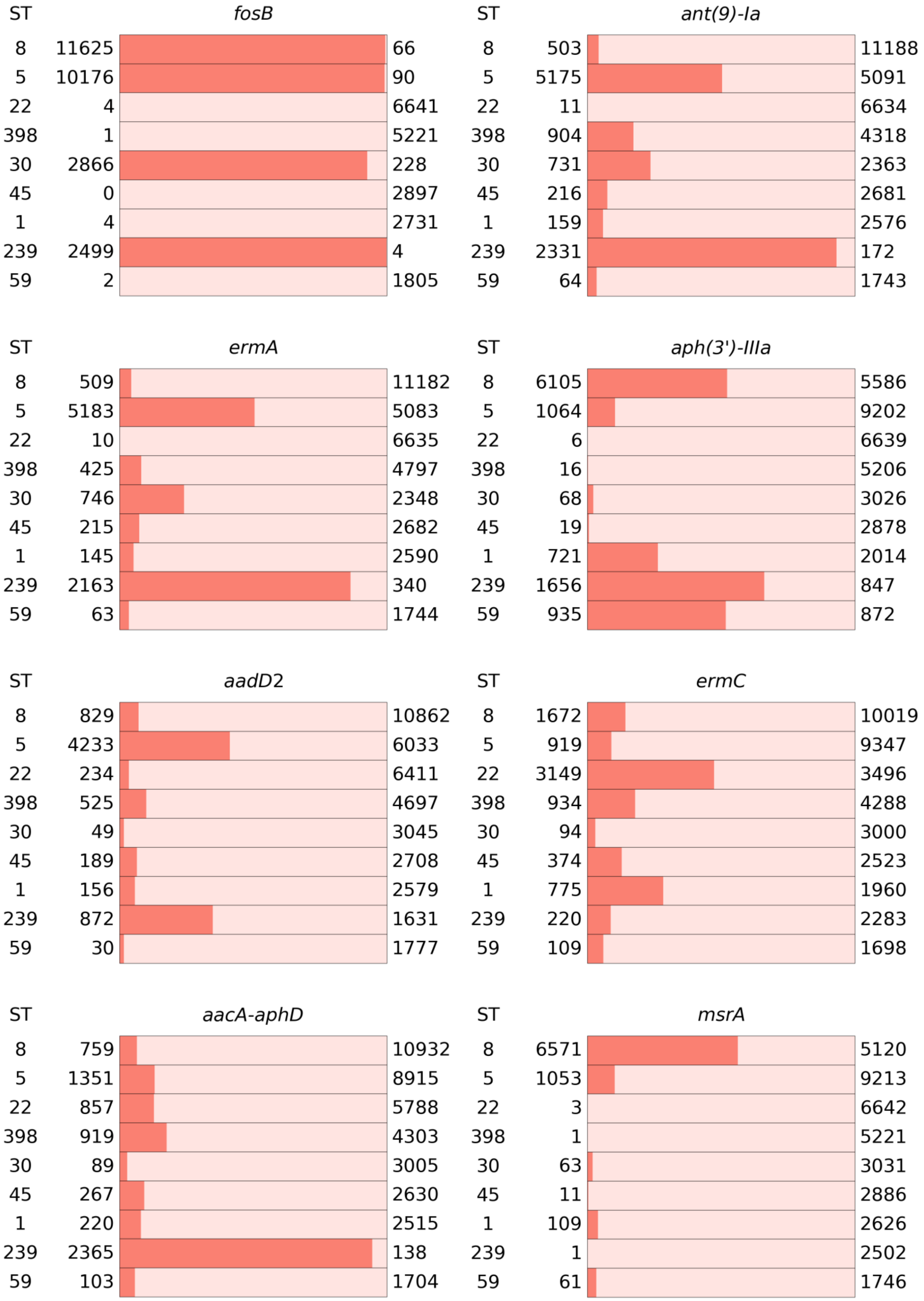

ST
fosB
8 11625 66
5 10176 90
22 4 6641
398 1 5221
30 2866 228
45 0 2897
1 4 2731
239 2499 4
59 2 1805
ST
ant(9)-Ia
8 503 11188
5 5175 5091
22 11 6634
398 904 4318
30 731 2363
45 216 2681
1 159 2576
239 2331 172
59 64 1743
ST
ermA
8 509 11182
5 5183 5083
22 10 6635
398 425 4797
30 746 2348
45 215 2682
1 145 2590
239 2163 340
59 63 1744
ST
aph(3')-IIIa
8 6105 5586
5 1064 9202
22 6 6639
398 16 5206
30 68 3026
45 19 2878
1 721 2014
239 1656 847
59 935 872
ST
aadD2
8 829 10862
5 4233 6033
22 234 6411
398 525 4697
30 49 3045
45 189 2708
1 156 2579
239 872 1631
59 30 1777
ST
ermC
8 1672 10019
5 919 9347
22 3149 3496
398 934 4288
30 94 3000
45 374 2523
1 775 1960
239 220 2283
59 109 1698
ST
aacA-aphD
8 759 10932
5 1351 8915
22 857 5788
398 919 4303
30 89 3005
45 267 2630
1 220 2515
239 2365 138
59 103 1704
ST
msrA
8 6571 5120
5 1053 9213
22 3 6642
398 1 5221
30 63 3031
45 11 2886
1 109 2626
239 1 2502
59 61 1746

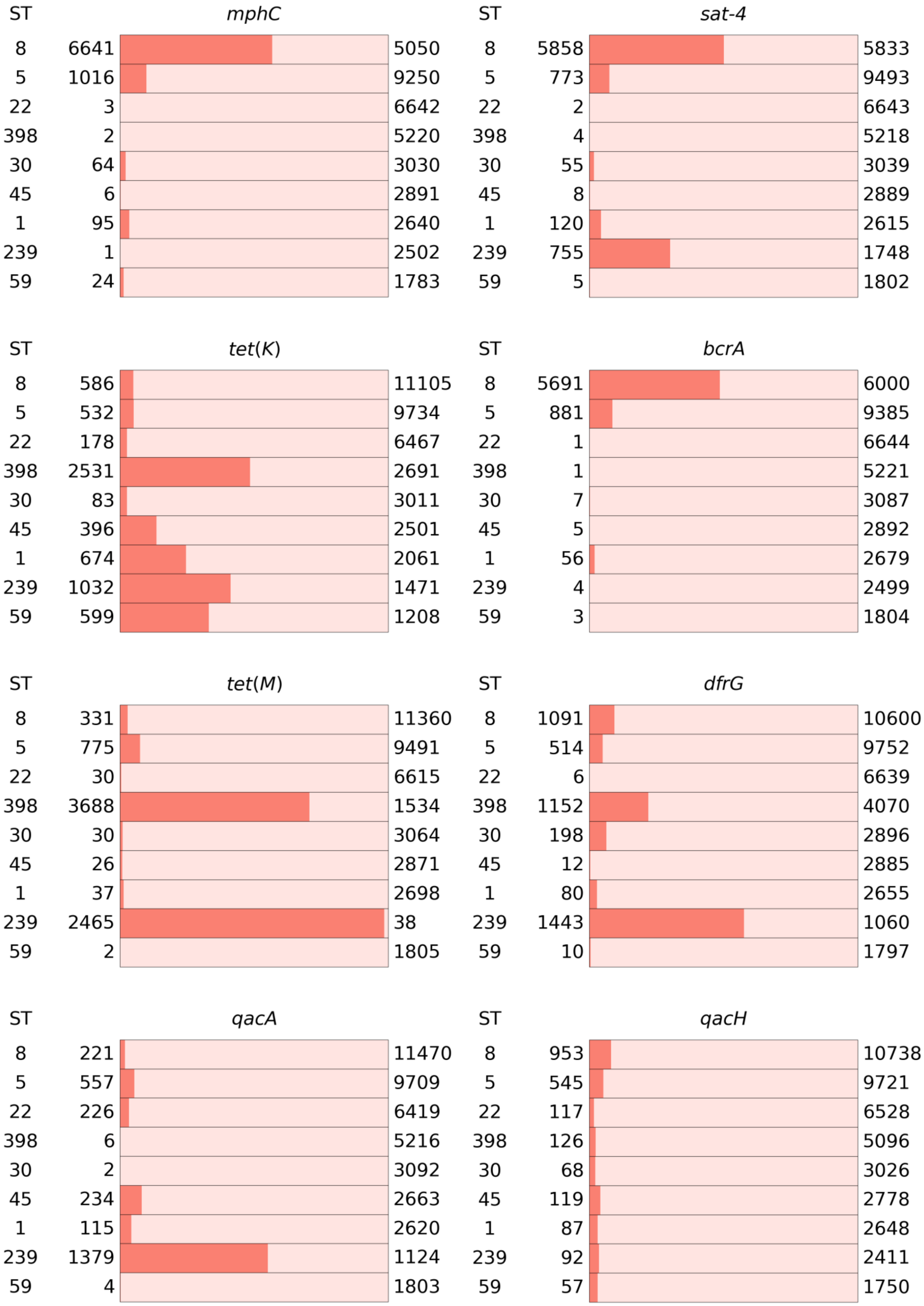
ST
mphC
8 6641 5050
5 1016 9250
22 3 6642
398 2 5220
30 64 3030
45 6 2891
1 95 2640
239 1 2502
59 24 1783
ST
sat-4
8 5858 5833
5 773 9493
22 2 6643
398 4 5218
30 55 3039
45 8 2889
1 120 2615
239 755 1748
59 5 1802
ST
tet(K)
8 586 11105
5 532 9734
22 178 6467
398 2531 2691
30 83 3011
45 396 2501
1 674 2061
239 1032 1471
59 599 1208
ST
bcrA
8 5691 6000
5 881 9385
22 1 6644
398 1 5221
30 7 3087
45 5 2892
1 56 2679
239 4 2499
59 3 1804
ST
tet(M)
8 331 11360
5 775 9491
22 30 6615
398 3688 1534
30 30 3064
45 26 2871
1 37 2698
239 2465 38
59 2 1805
ST
dfrG
8 1091 10600
5 514 9752
22 6 6639
398 1152 4070
30 198 2896
45 12 2885
1 80 2655
239 1443 1060
59 10 1797
ST
qacA
8 221 11470
5 557 9709
22 226 6419
398 6 5216
30 2 3092
45 234 2663
1 115 2620
239 1379 1124
59 4 1803
ST
qacH
8 953 10738
5 545 9721
22 117 6528
398 126 5096
30 68 3026
45 119 2778
1 87 2648
239 92 2411
59 57 1750

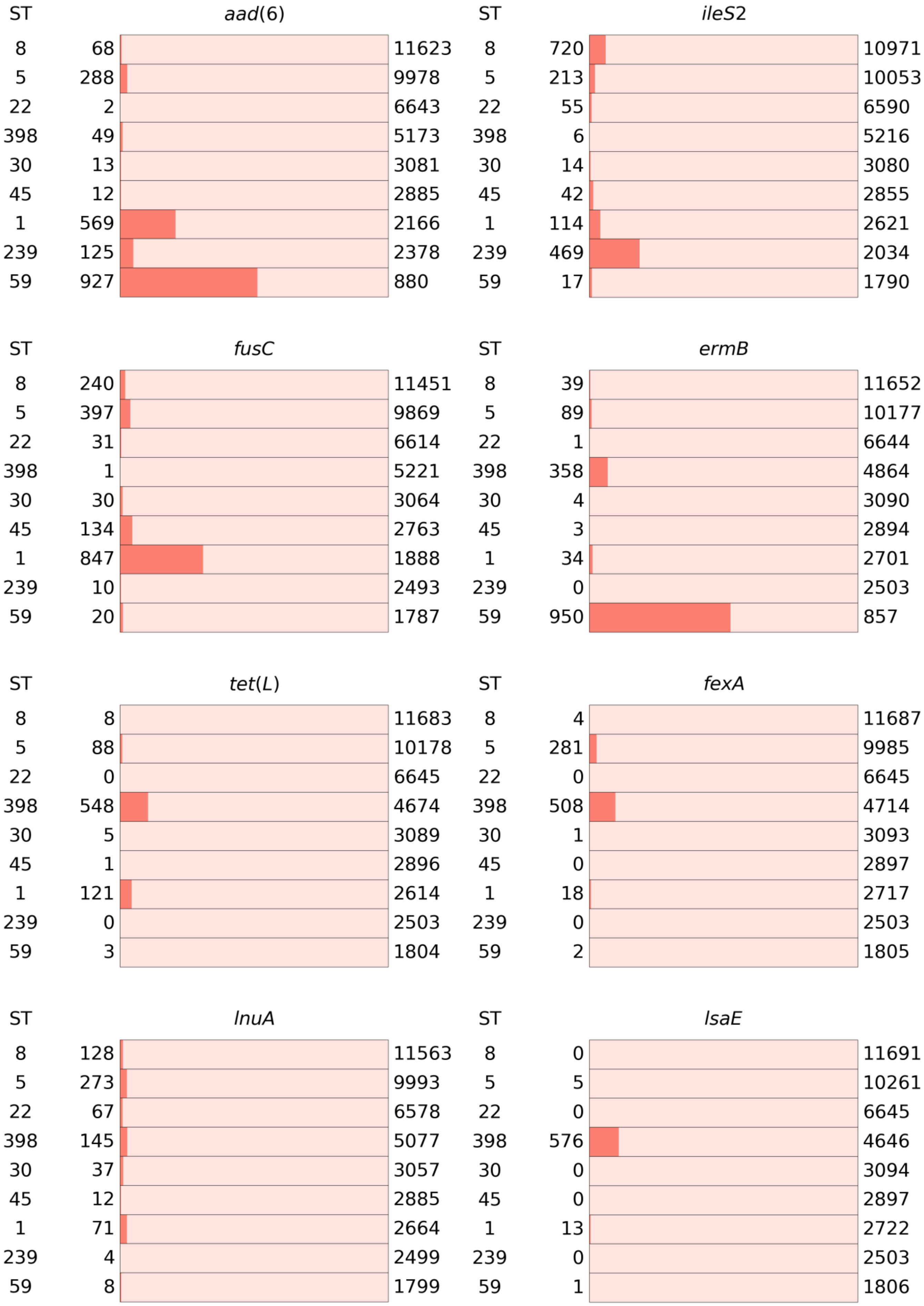

ST
aad(6)
8 68 11623
5 288 9978
22 2 6643
398 49 5173
30 13 3081
45 12 2885
1 569 2166
239 125 2378
59 927 880
ST
ileS2
8 720 10971
5 213 10053
22 55 6590
398 6 5216
30 14 3080
45 42 2855
1 114 2621
239 469 2034
59 17 1790
ST
fusC
8 240 11451
5 397 9869
22 31 6614
398 1 5221
30 30 3064
45 134 2763
1 847 1888
239 10 2493
59 20 1787
ST
ermB
8 39 11652
5 89 10177
22 1 6644
398 358 4864
30 4 3090
45 3 2894
1 34 2701
239 0 2503
59 950 857
ST
tet(L)
8 8 11683
5 88 10178
22 0 6645
398 548 4674
30 5 3089
45 1 2896
1 121 2614
239 0 2503
59 3 1804
ST
fexA
8 4 11687
5 281 9985
22 0 6645
398 508 4714
30 1 3093
45 0 2897
1 18 2717
239 0 2503
59 2 1805
ST
lnuA
8 128 11563
5 273 9993
22 67 6578
398 145 5077
30 37 3057
45 12 2885
1 71 2664
239 4 2499
59 8 1799
ST
lsaE
8 0 11691
5 5 10261
22 0 6645
398 576 4646
30 0 3094
45 0 2897
1 13 2722
239 0 2503
59 1 1806

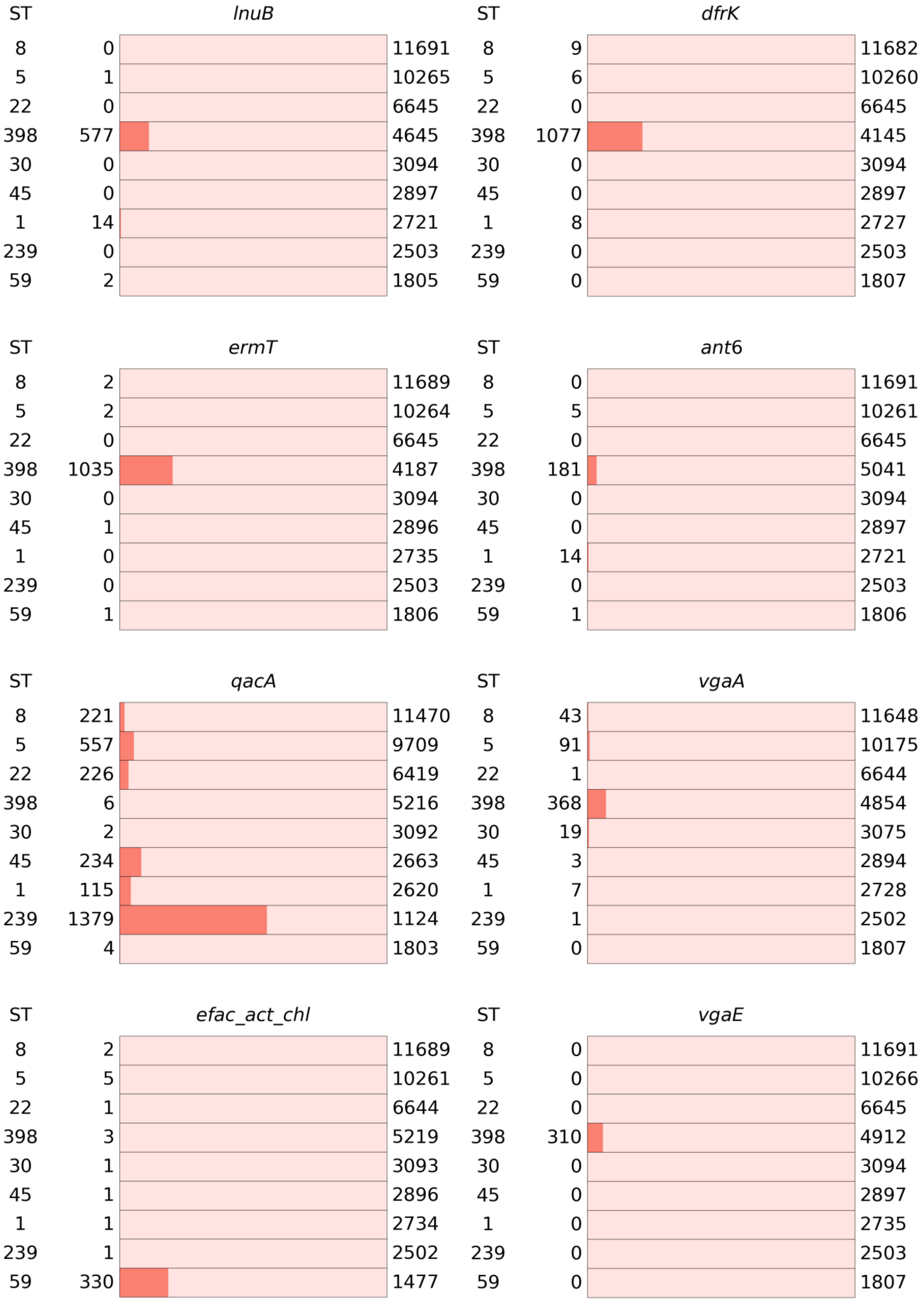

ST
lnuB
8 0 11691
5 1 10265
22 0 6645
398 577 4645
30 0 3094
45 0 2897
1 14 2721
239 0 2503
59 2 1805
ST
dfrK
8 9 11682
5 6 10260
22 0 6645
398 1077 4145
30 0 3094
45 0 2897
1 8 2727
239 0 2503
59 0 1807
ST
ermT
8 2 11689
5 2 10264
22 0 6645
398 1035 4187
30 0 3094
45 1 2896
1 0 2735
239 0 2503
59 1 1806
ST
ant6
8 0 11691
5 5 10261
22 0 6645
398 181 5041
30 0 3094
45 0 2897
1 14 2721
239 0 2503
59 1 1806
ST
qacA
8 221 11470
5 557 9709
22 226 6419
398 6 5216
30 2 3092
45 234 2663
1 115 2620
239 1379 1124
59 4 1803
ST
vgaA
8 43 11648
5 91 10175
22 1 6644
398 368 4854
30 19 3075
45 3 2894
1 7 2728
239 1 2502
59 0 1807
ST
efac_act_chl
8 2 11689
5 5 10261
22 1 6644
398 3 5219
30 1 3093
45 1 2896
1 1 2734
239 1 2502
59 330 1477
ST
vgaE
8 0 11691
5 0 10266
22 0 6645
398 310 4912
30 0 3094
45 0 2897
1 0 2735
239 0 2503
59 0 1807

ST	*mecC-type_blaZ*	
8	0	11691
5	2	10264
22	0	6645
398	0	5222
30	0	3094
45	0	2897
1	2	2733
239	0	2503
59	0	1807

ST	*mecC*	
8	2	11689
5	1	10265
22	0	6645
398	0	5222
30	0	3094
45	0	2897
1	1	2734
239	1	2502
59	0	1807

ST	*apmA*	
8	0	11691
5	1	10265
22	0	6645
398	114	5108
30	0	3094
45	0	2897
1	0	2735
239	0	2503
59	0	1807

ST	*spd*	
8	1	11690
5	1	10265
22	0	6645
398	87	5135
30	1	3093
45	0	2897
1	0	2735
239	1	2502
59	0	1807

ST	*cfrA*	
8	2	11689
5	8	10258
22	0	6645
398	55	5167
30	1	3093
45	0	2897
1	5	2730
239	0	2503
59	0	1807

ST	*vgaALC*	
8	4	11687
5	4	10262
22	0	6645
398	31	5191
30	0	3094
45	0	2897
1	0	2735
239	0	2503
59	0	1807

ST	*fexB*	
8	1	11690
5	1	10265
22	0	6645
398	2	5220
30	0	3094
45	0	2897
1	1	2734
239	0	2503
59	1	1806

Fig. S2. Distribution of type II toxin-antitoxin (TA) systems and drug resistance (R) determinants among *Staphylococcus aureus* genomes belonging to the top 9 most numerous STs. Overall, even less prevalent TA systems and R determinants tend to occur in more than one ST. However, TA/R pairs of strongly negative co-occurrence tend to be dominant in different lineages, suggesting their incompatibility. The numbers next to bar charts indicate exact numbers of genomes containing a given element (dark orange) or devoid of it (light orange). TA and R ordered descending with respect to the occurrence.

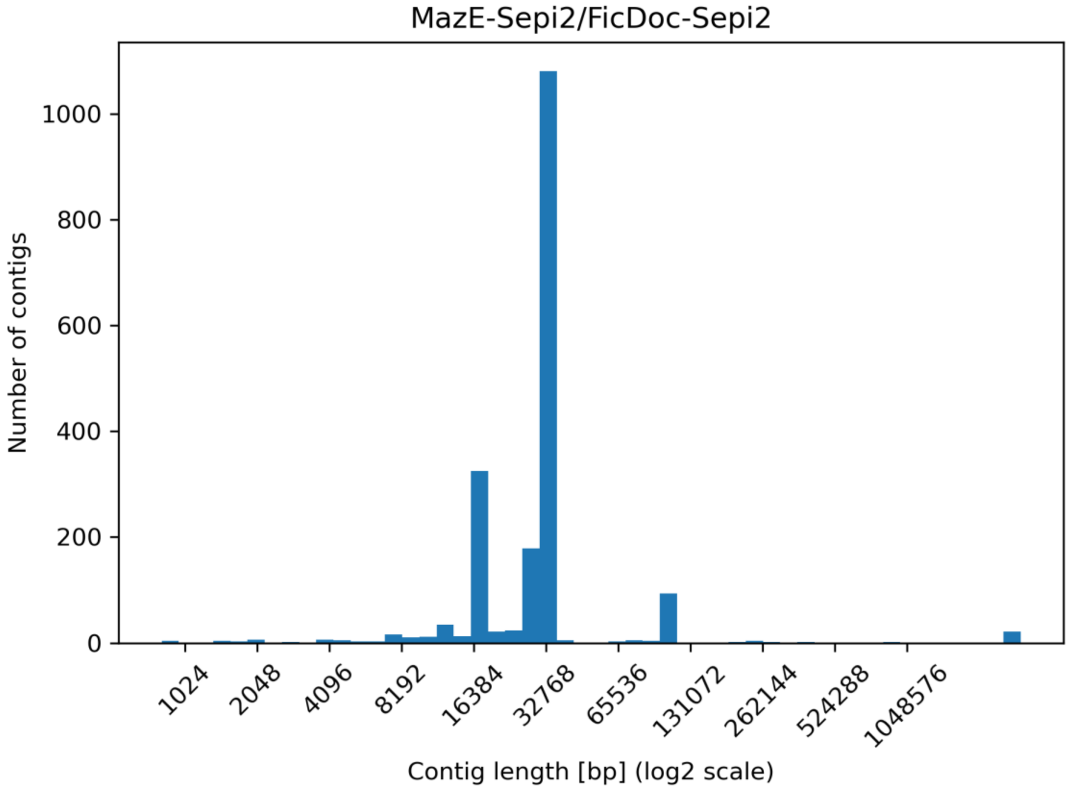

Fig. S3. Length distribution of MazE-Sepi/FicDoc-Sepi2-carrying contigs. In clear majority of cases the system is localised in contigs of length c. 32.7 and 16.4 kbp.

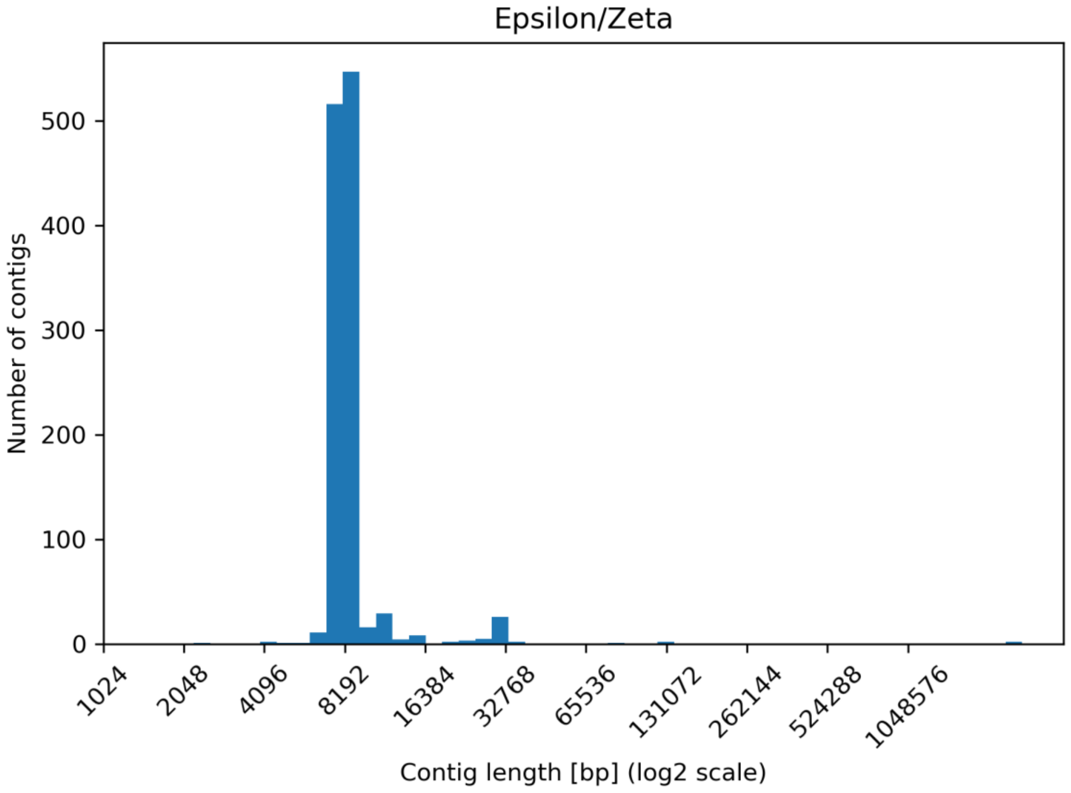

Fig. S4. Length distribution of Epsilon/Zeta-carrying contigs. In clear majority of cases the system is localised in contigs of length c. 8.2 kbp.

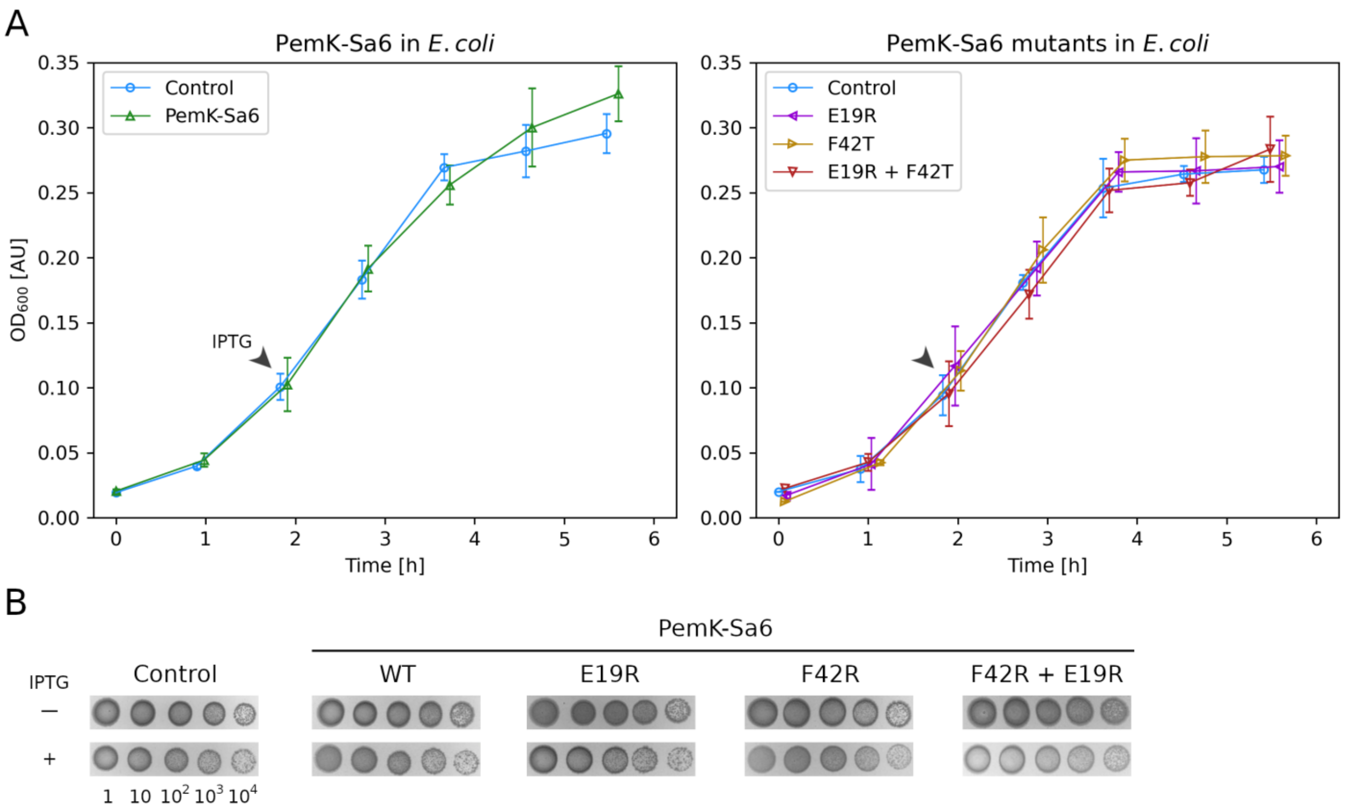

Fig. S5. Impact of PemK-Sa6 and its mutants on *E. coli* growth. (**A**) Cadmium-dependent PemK-Sa6 expression in *S. aureus* does not inhibit the liquid culture growth (chart on the left). PemK-Sa6 E19R and F42T mutations in the catalytic site do not restore any toxic activity towards bacterial growth, neither separately nor jointly (chart on the right). The arrow points to the time of IPTG introduction to the culture. (**B**) Similar observations are made when bacteria are grown on solid media.

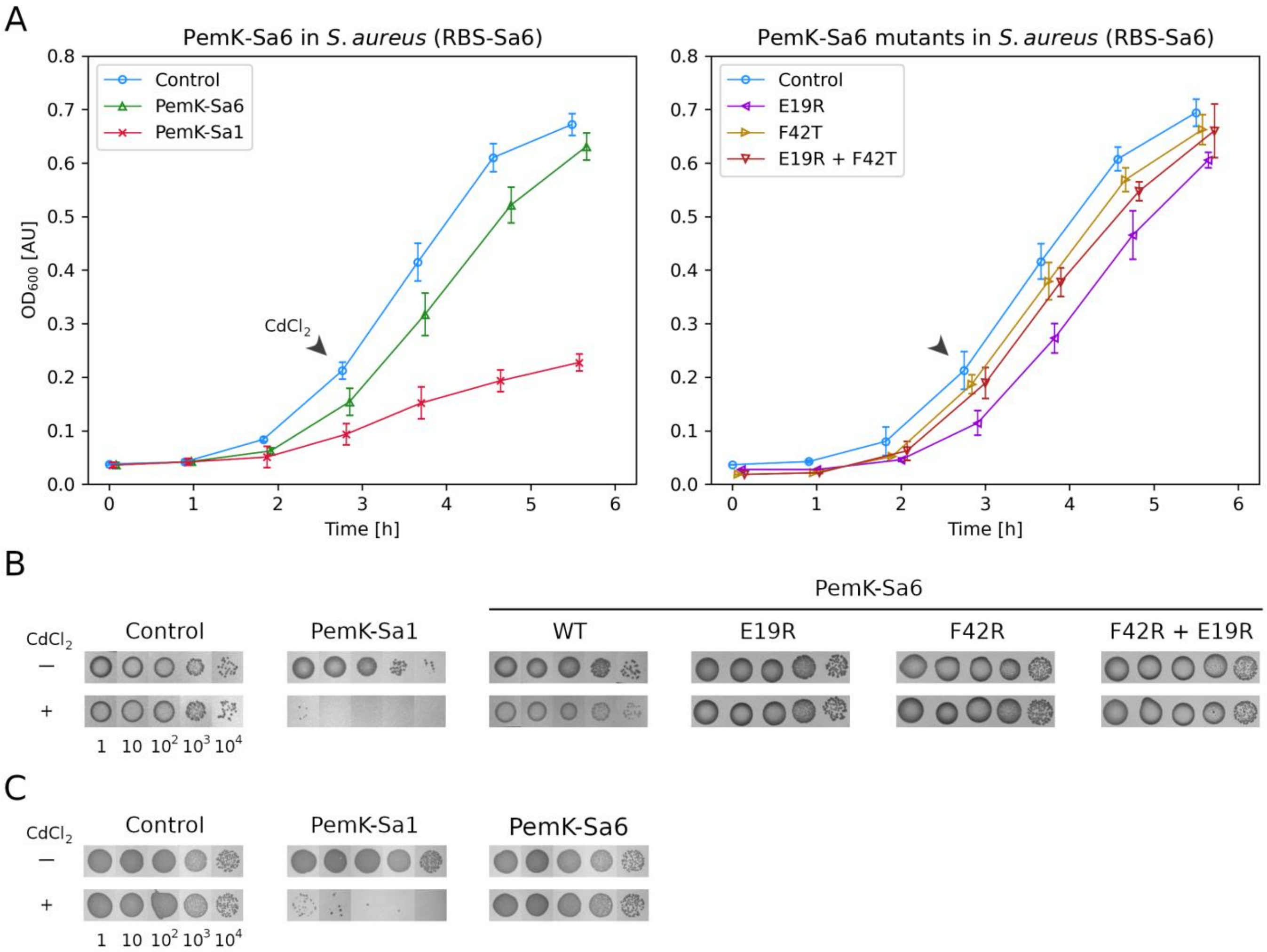

Fig. S6. Impact of PemK-Sa6 and its mutants expressed with the toxin native RBS site on *S. aureus* and *S. pseudintermedius* growth. (**A**) Cadmium-dependent PemK-Sa6 expression in *S. aureus* does not inhibit the liquid culture growth, as does PemK-Sa1 (chart on the left). PemK-Sa6 E19R and F42T mutations in the catalytic site do not restore any toxic activity towards bacterial growth, separately or jointly (chart on the right). The arrow points to the time of $CdCl_2$ introduction to the culture. (**B**) Similar observations are made when bacteria are grown on solid media. (**C**) Comparative results on growth inhibition on solid media by PemK-Sa1 and PemK-Sa6 in *S. pseudintermedius*, the native carrier of PemIK-Sa6 system.

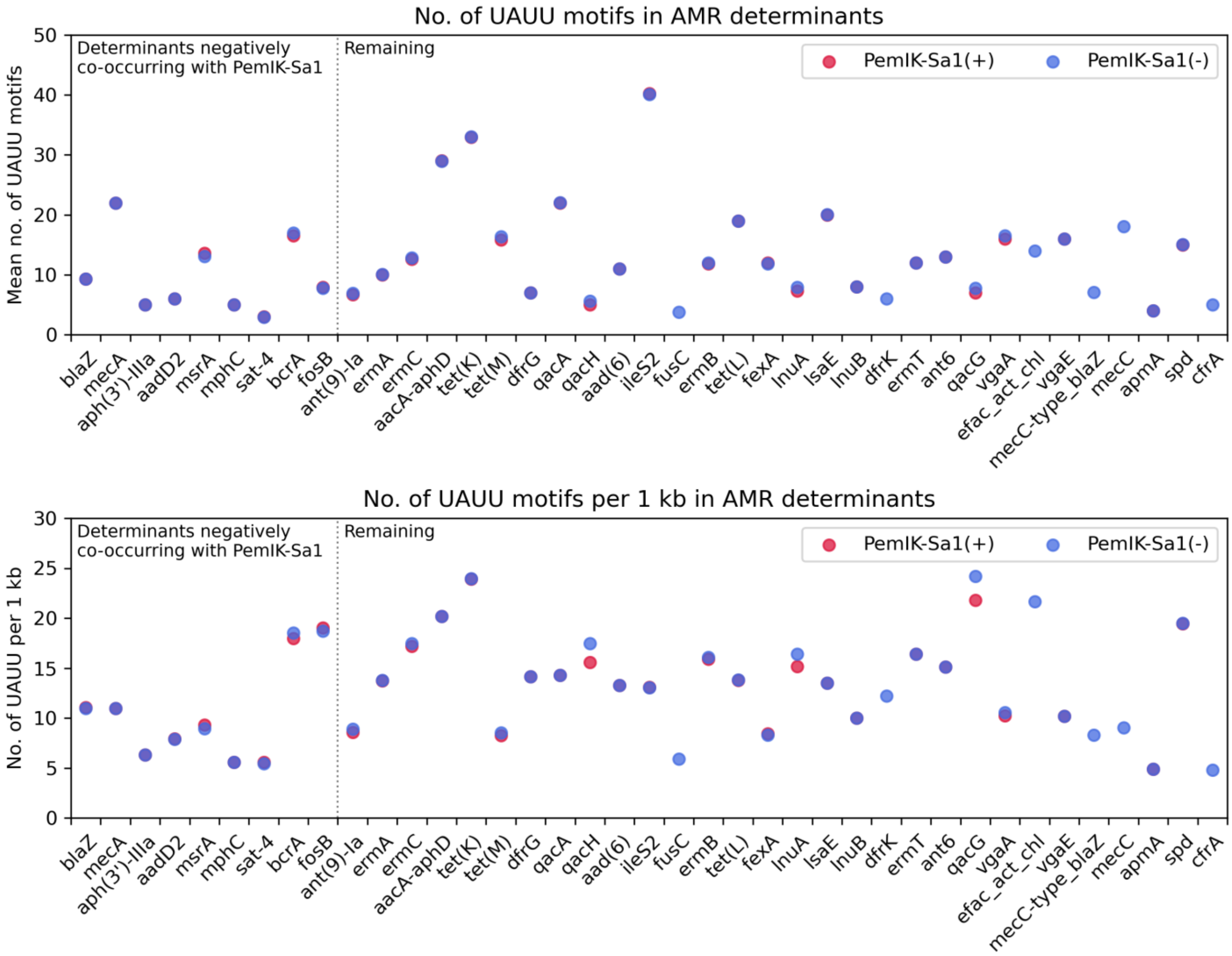

Fig. S7. Occurrence of UAUU motif recognised by PemK-Sa1 in transcripts of different AMR determinants expressed as a mean number of motifs per transcript (upper plot) and mean number of motifs per 1 kb of a transcript (lower plot). Standard deviation values were negligibly low and were not depicted. The order of AMR determinants corresponds to Fig. 1. of the main manuscript. However, AMRs negatively correlating with PemIK-Sa1 are grouped first (before the dashed line). The statistics of UAUU motif occurrence are not different between transcripts co-occurring (PemIK-Sa1(+)) and non-occurring (PemIK-Sa1(-)) with PemIK-Sa1.

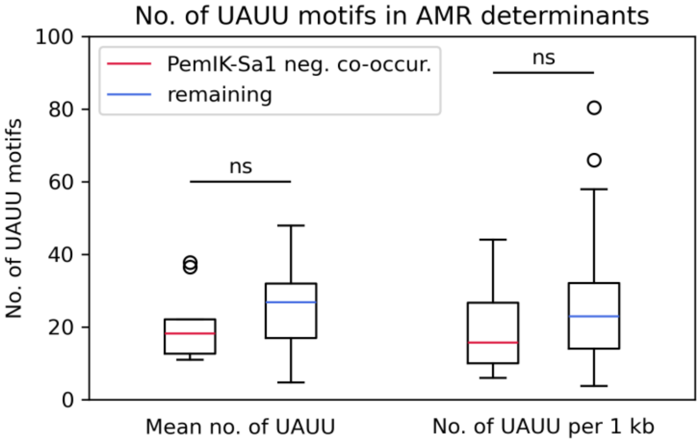

Fig. S8. Occurrence of UAUU motif recognised by PemK-Sa1 in transcripts of different AMR determinants expressed as a mean number of motifs per transcript (upper plot) and mean number of motifs per 1 kb of a transcript (lower plot). The data were analysed collectively and the distribution of UAUU motif among transcripts co-occurring and non-occurring with PemIK-Sa1 was tested using the Mann-Whitney U test. No significant differences are observed (ns).

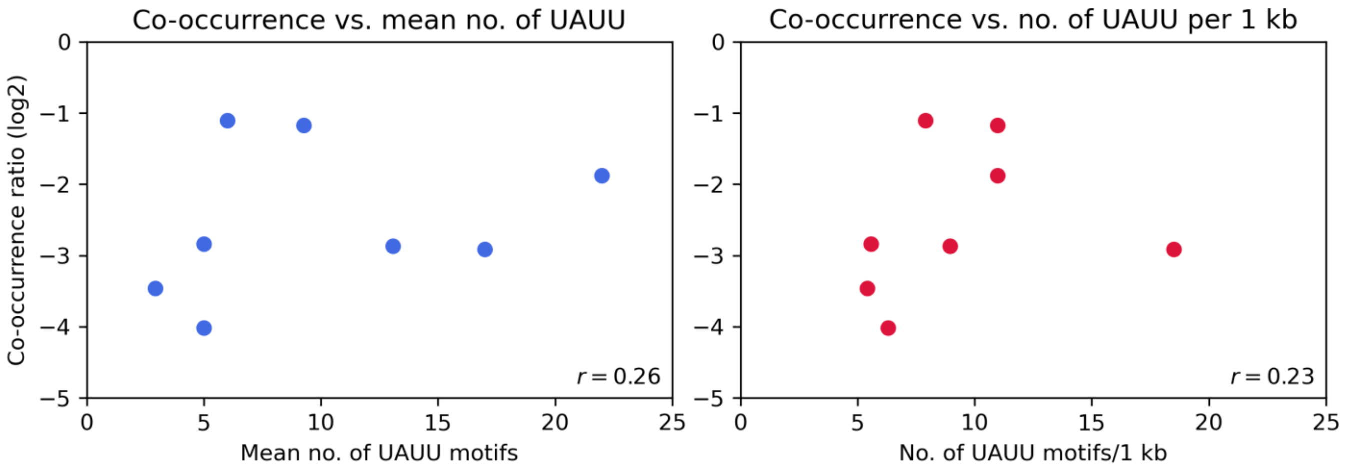

Fig. S9. Relation between UAUU motif occurrence and co-occurrence ratios for transcripts negatively co-occurring with PemIK-Sa1. The occurrence of the UAUU motif was expressed as a mean number of motifs per transcript (left plot) and mean number of motifs per 1 kb of a transcript (right plot). In both cases no relation is observed as evidenced by low Pearson correlation coefficients (*r*).

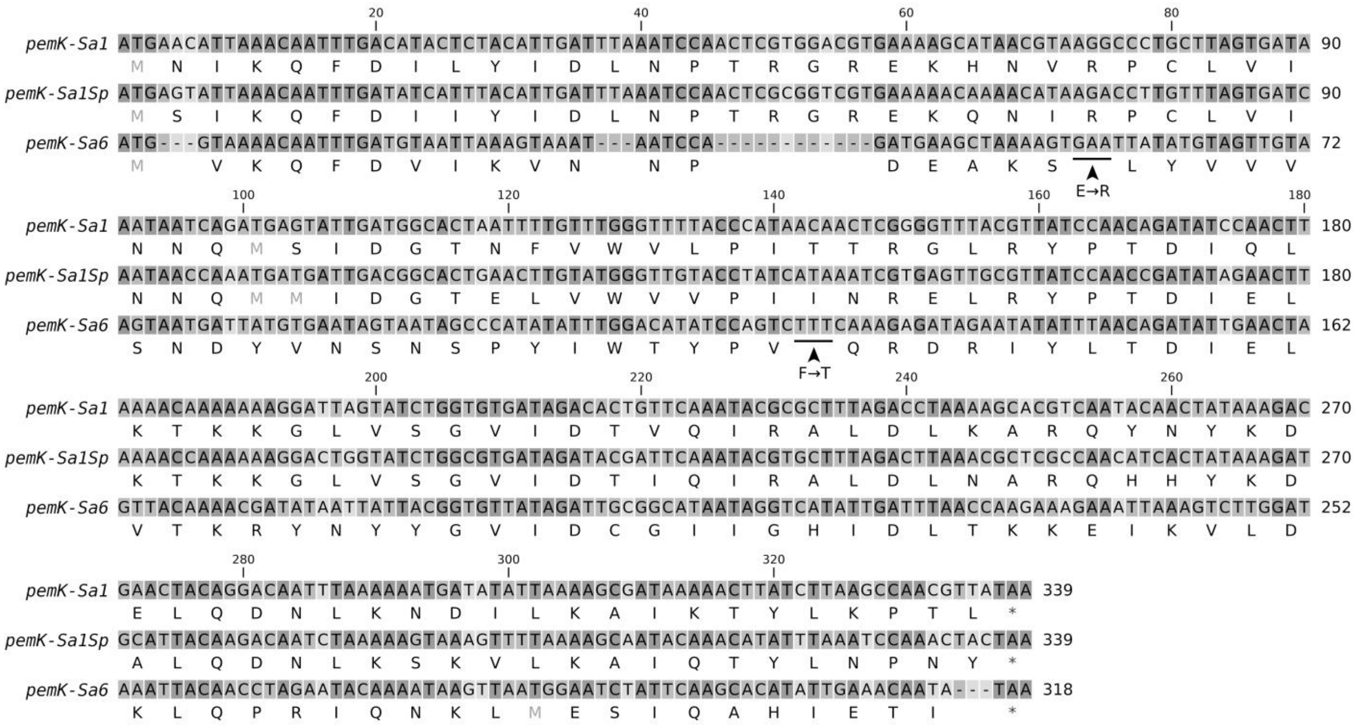

Fig. S10. Coding sequences of PemK-Sa1, -Sa1Sp, and -Sa6. The coding sequences of PemK-Sa1 and -Sa1Sp are largely identical, sharing 79.1% sequence identity (78.6% identity and 88.4% similarity for protein sequences). In contrast, the PemK-Sa6 coding sequence differs significantly from the other two, with sequence identities, respectively, of 46.0% (27.7% identity and 54.5% similarity for protein sequences) and 48.1% (30.4% identity and 54.5% similarity for protein sequences). Mutation sites in PemK-Sa6 are indicated under the multiple sequence alignment. This alignment corresponds to the protein sequence alignment presented in the original publication.
