# Supplementary material for: Analysis of co-occurrence of type II toxin–antitoxin systems and antibiotic resistance determinants in Staphylococcus aureus
Source: mSystems. 2025 Feb 27;10(3):e00957-24. doi: 10.1128/msystems.00957-24 (PMC11915791; doi:10.1128/msystems.00957-24)
Supplement: Supplemental material — Information for Table S1 and drug resistance names. [file msystems.00957-24-s0005.pdf]

Supplementary table S1: Distribution of TA systems and resistance determinants (drugs) in all analysed *Staphylococcus aureus* genomes. If possible to determine, for each genome MLST type and clonal complex are provided.

Supplementary table S1 is available here:

https://doi.org/10.57903/UJ/M38YMS

Synonymous drug resistance names used in the publication:

aadD2	– ANT(4')-Ib
aacA-aphD	– AAC6_Ie_APH2_Ia
ileS2	– Saur_mupA_MUP
ant6	– ANT(6)-Ia
